# *Slc6a9* is distributed in glial cells and neurons across several nervous system regions, whereas *Slc6a5* is more restricted to neurons in the caudal brain

**DOI:** 10.1186/s12868-026-00999-3

**Published:** 2026-02-17

**Authors:** Mikaela M. Ceder, Malin C. Lagerström

**Affiliations:** Department of Immunology, Genetics and Pathology, Uppsala University, Uppsala, Sweden

**Keywords:** GLYT1, *Slc6a9*, GLYT2, *Slc6a5*, Excitatory, Inhibitory, Sex-dependent differences

## Abstract

**Background:**

The glycinergic system constitutes a main source of inhibitory regulation in the central nervous system. Glycine transporters (GLYT1 and GLYT2), encoded by *Slc6a9* and *Slc6a5*, respectively, are responsible for glycine reuptake and clearance from the synaptic cleft, thereby maintaining neurotransmitter homeostasis. Emerging evidence from pharmacological and mechanistic studies has highlighted GLYTs as promising therapeutic targets for psychiatric disorders and persistent pain. Nevertheless, data on anatomical and cellular distribution of GLYTs and sex-dependent differences in GLYT expression remain limited.

**Methods:**

To address this gap, the aim of this study was to examine the *Slc6a9* and *Slc6a5* mRNA expression across mouse brain regions and peripheral organs using three complementary approaches focusing on mRNA expression: re-analysis of single-cell RNA sequencing data, quantitative RT-PCR, and RNAscope.

**Results:**

Both genes were detected in multiple brain regions, with *Slc6a9* exhibiting a broader distribution in both glial cells and neurons, while *Slc6a5* was more restricted to neurons. Sex-dependent differences were detected for *Slc6a9* in the amygdala and thalamus, liver, intestine, spleen, kidney and genitalia using quantitative RT-PCR, and for *Slc6a5* in the cortex, striatum, hippocampus, and spinal cord using quantitative RT-PCR. Spatial analysis of the glycine transporters showed that *Slc6a9* can be found in several brain regions spanning the rostral to the caudal axis, in both glial cells and neurons, while *Slc6a5* was more restricted to the caudal brain regions.

**Conclusions:**

In general, in regions where differences were detected using quantitative RT-PCR, higher expression levels were observed in male mice. Moreover, *Slc6a9* expression was found to occur in both glial cells, such as astrocytes, oligodendrocytes and ependymal cells, as well as both excitatory and inhibitory neurons, while *Slc6a5* mainly occurred in inhibitory neurons. These findings provide novel insights into the spatial and sex-dependent expression of glycine transporters.

**Supplementary Information:**

The online version contains supplementary material available at 10.1186/s12868-026-00999-3.

## Introduction

The glycinergic system serves a dual role as both a major source of inhibitory signalling [1–3] and an essential excitatory modulator [4–7] in the central nervous system. Glycine is packaged into synaptic vesicles by the vesicular inhibitory amino acid transporter (VIAAT) [8], and released from presynaptic terminals following depolarization. Upon release, glycine binds to chloride-permeable pentameric ligand-gated ion channels, leading to hyperpolarization of the postsynaptic neuron [1–3]. Furthermore, in brain regions like the cortex and hippocampus, glycine acts as a N-methyl-D-aspartate (NMDA) receptor co-agonists, where it is required alongside glutamate to enable e.g., excitatory neurotransmission and synaptic plasticity [4–7]. Reuptake and clearance from the synaptic cleft are subsequently mediated by glycine transporters (GLYT1 and GLYT2), ensuring neurotransmitter homeostasis [9, 10]. Together, these mechanisms enable complex processes such as motor and sensory functions [11–13], as well as learning and memory formation [13–16].

The GLYTs belong to the sodium- and chloride-dependent neurotransmitter transporter (SLC6) family of solute carriers [17] and are broadly conserved across vertebrates [18]. GLYT1, later designated as *Slc6a9*, was first identified and characterized in mice in the early 1990s [19]. In humans, GLYT1 has been associated with essential hypertension [20], and adolescent idiopathic scoliosis [21], while genetic knockout of GLYT1 in mice leads to lethality within 12 h of birth [22]. Furthermore, mutations in the *GLYT1* gene results in Glycine transporter 1 encephalopathy, identified by startle-like responses, respiratory failure and severe hypotonia during early infancy [23]. Polymorphisms of *SLC6A9* have been associated with psychiatric disorders such as schizophrenia and major depressive disorder [24–27], showcasing that the glycine signalling has other key functions beyond its role in hyperpolarization in the central nervous system. GLYT1 is predominantly expressed in glia cells, and in the brain, *SLC6A9* is distributed across multiple regions, including the cerebral cortex, olfactory bulb, hippocampal formation, basal ganglia, thalamus, hypothalamus, midbrain, pons, medulla oblongata, cerebellum, pituitary gland, retina and white matter in humans, pigs, and mice [28], as well as in the developing mouse spinal cord [29]. These expression patterns are largely consistent with the mapping of *Slc6a9* mRNA expression generated by the Allen Brain Institute (https://mousespinal.brain-map.org/imageseries/show.html?id=100069899, https://mouse.brain-map.org/gene/show/14440) [30, 31].

GLYT2 was first identified in rodents in 1993 and is abundantly expressed in caudal regions of the brain and the spinal cord [32]. Knockout of GLYT2 in mice, resulting in reduced glycine transport in the brainstem and spinal cord, produces a lethal phenotype similar to human hyperekplexia [33]. In mice, *Slc6a5* expression is prominent in the cerebellum, pons, medulla oblongata and spinal cord (https://mousespinal.brain-map.org/imageseries/detail/100024105.html, https://mouse.brain-map.org/gene/show/68410) [30, 31], as well as the pontine reticular formation and periaqueductal grey (PAG) [20, 21], with lower levels detected in the midbrain [28, 29]. Moreover, pharmacological inhibition of GLYT2 have been demonstrated to ameliorate mechanical allodynia [34]. Emerging evidence from studies on transport mechanisms, cellular pathways, and pharmacological interventions, particularly in psychiatric disorders and chronic pain, highlights the importance of GLYTs as potential therapeutic targets, where GLYT1 and GLYT2 inhibitors would potentiate the modulatory role of glycine in these neuronal circuits [35–37]. Despite this, data on sex-dependent differences in GLYT expression remains scarce. Considering the well-established sex differences in psychiatric disorders and pain [38–41], understanding these variations could aid the prediction of adverse effects. To address this gap, we investigated the mRNA expression of *Slc6a9* and *Slc6a5* across the mouse nervous system and peripheral organs using different complementary mRNA-based approaches: re-analysis of a single-cell RNA sequencing (scRNA-seq) dataset [42], quantitative RT-PCR (qRT-PCR), and RNAscope. Furthermore, sex-dependent differences in mRNA expression were assessed using qRT-PCR.

## Methods

### Pre-processing of Zeisel et al. (2018) single-cell RNA sequencing dataset

The expression of *Slc17a6* (vesicular glutamate transporter 2), *Slc32a1* (vesicular inhibitory amino acid transporter), *Slc6a9* and *Slc6a5* in neurons in the nervous system was analysed using the scRNA-seq ‘15all.loom’ dataset [42], obtained from http://linnarssonlab.org/ as previously described [43, 44]. The original Zeisel et al. dataset comprises expression profiles of 27,998 genes across 160,796 single cells derived from wild-type outbred strains CD-1 (Charles River) and Swiss (Janvier), as well as Vgat-Cre; tdTomato mice to target inhibitory neurons, and Wnt1-Cre; R26Tomato mice to isolate neurons from the peripheral and enteric nervous systems [42]. Data analysis was performed using SCANPY 1.9.1 [45] in Python 3.8.8, with the full code detailed in https://github.com/HannahMWeman/glra3-expression-analysis-in-the-nervous-system [43, 44, 46]. To assess the expression of *Slc6a9* and *Slc6a5* in glial cells, as well as investigating *Slc6a9* or *Slc6a5* co-expressing both *Slc17a6* and *Slc32a1* (triple positive cells), the code can be found at https://github.com/MikaelaCeder/analysis-glycine-transporter-expression.ipynb. The prevalence of *Slc6a5* and *Slc6a9* expression (defined as log1p > 0.1) was calculated for each nervous system region as described in [43]. Furthermore, the expression patterns of *Slc17a6* and *Slc32a1*, well-established markers for excitatory and inhibitory neurons [47, 48], respectively, were examined in all *Slc6a5*- and *Slc6a9*-positive neurons (833 and 3,076 neurons, respectively) across the different nervous system regions using visualization and occurrence-based analyses.

### Animals

All animal procedures were approved by the regional animal research ethics committee (Uppsala djurförsöksetiska nämnd) and conducted in accordance with the Swedish Animal Welfare Act (SFS 2018:1192), the Swedish Animal Welfare Ordinance (SFS 2019:66), and the Regulations and General Advice for Laboratory Animals (SJVFS 2019:9, Saknr L 150) (permit numbers C419/12, C39/16, and 5.8.18–01428/2023). Reporting followed the ARRIVE guidelines 2.0 Essential 10, where applicable. Both female and male C57BL/6J mice (Taconic, Denmark) were included in the study. Mice were housed with littermates in individually ventilated cages (floor area ~ 501 cm²; maximum five mice per cage) containing bedding and environmental enrichment. Housing conditions were maintained at 20–24 °C with 45–65% humidity under a 12-h light/dark cycle (lights on at 06:00). Food (diet pellets, Ssniff, Sweden) and tap water were provided ad libitum. All procedures were designed to minimize stress, and euthanasia was performed during the light phase.

### Quantitative RT-PCR

Tissues from five adult male C57BL/6J mice (10–14 weeks) were previously collected and processed as described in [49, 50]. In addition, tissues from five adult females (14 weeks) and five adult males (10–11 weeks) were collected, prepared, and analysed according to [43]. In short, the C57BL/6J mice were euthanized via cervical dislocation, without prior treatment, during the light period. Total RNA was isolated using the Absolutely RNA Mini Kit (Qiagen, Germany) and converted to cDNA with the High-Capacity RNA-to-cDNA Kit (Applied Biosystems, Invitrogen, USA), following the protocols outlined in [43].

Primers were designed using the ThermoFisher Scientific primer design tool, which is based on the Primer3 algorithm [51] or Beacon Design 8 (Premier Biosoft) for the reference genes *Actb*, *Rpl19*, *Cyclo* and *Gapdh*. All primer sequences were subsequently screened using BLAST and global alignment analyses [52] to minimize the risk of non-specific amplification. *Slc6a5* forward 5′-*tgcggccactcagattttct*-3′, reverse 5′-*tgttgactttgcgctcgttg*-3′; *Slc6a9* forward 5′-*tgaccaccactgctcatgtc*-3′, reverse 5′-*agattttcctgggcagaggc*-3′. Reference housekeeping genes: actin-related protein 1B (*Actb*) forward 5′-*ccttcttgggtatggaatcctgtg*-3′, reverse 5′-*cagcactgtgttggcatagagg*-3′; ribosomal protein L19 (*Rpl19*) forward 5′-*aatcgccaatgccaactc*-3′, reverse 5′-*ggaatggacagtcacagg*-3′; Peptidylprolyl isomerase A (*Cyclo*) forward 5′-*tttgggaaggtgaaagaagg*-3′, reverse 5′-*acagaaggaatggtttgatgg*-3′ and glyceraldehyde-3-phosphate dehydrogenase (*Gapdh*) forward 5′-*gccttccgtgttcctacc*-3′, reverse 5′-*gcctgcttcaccaccttc*-3.

Expression of *Slc6a5* and *Slc6a9* was quantified using qRT-PCR in a final reaction volume of 20 µl, as previously described [43, 44]. Reactions were performed on an iCycler real-time detection system (Bio-Rad, USA) under the following cycling conditions: initial denaturation at 95 °C for 30 s, followed by 45 cycles of 95 °C for 10 s, annealing at 55 °C for reference genes, 56 °C for *Slc6a5*, or 62 °C for *Slc6a9* for 30 s, and extension at 72 °C for 30 s. Melting curve analysis was conducted by increasing the temperature from 55 to 95 °C in 0.5 °C increments with a 10 s dwell time and plate read at each step. All reactions were run in triplicate, with a negative control included on each plate. Cycle threshold (Ct) values were obtained using CFX Maestro software (Bio-Rad, USA), and primer efficiencies were calculated with LinRegPCR.

### In situ hybridization and imaging

Tissue from four C57BL/6J mice (two females and two males, 10–14 weeks old) was prepared as described in [43, 44]. In short, the C57BL/6J mice were intraperitoneally injected with 0.2 to 0.3 ml (1:1) Ketamine (Ketalar, 50 mg/ml, Pfizer, Sweden) and Medetomidine (Domitor, 1 mg/ml, Orion Pharma, Sweden) and subsequently perfused with autoclaved ice-cold 1 × PBS. To minimize the risk of contamination and altered gene expression, the following steps were performed as quickly as possible in autoclaved ice-cold 1 × PBS; the whole brains and all divisions of the spinal cord were dissected and cleaned from meninges, followed by embedding in optimal cutting temperature (OCT) medium (Bio-Optica, Italy) and snap-frozen on dry ice in -80 °C isopentane (Sigma-Aldrich, Germany). Sections for female and male mice were chosen from anatomically matched regions, but minor differences in sectioning angle or tissue curvature might lead to slight visual shifts.

Fluorescent in situ hybridization was performed to detect the expression of *Slc6a5* in combination with *Slc17a6* and *Slc32a1*, or *Slc6a9* in brain and spinal cord using the RNAscope Fluorescent Multiplex kit (cat. #323270, ACD, USA), following ACD guidelines for formaldehyde-fixed and frozen tissues and as described in [44]. The following probes were used: *Slc6a5* (409741-C3), *Slc6a9* (525151-C1), *Slc17a6* (319171-C2), and *Slc32a1* (319191-C2).

RNAscope was also combined with immunohistochemistry on a set of slides. Briefly, FISH was performed as above, except the protease IV step was limited to 5 min, and antigen retrieval was carried out according to ACD guidelines. Slides were blocked in Supermix (0.25% gelatine and 0.5% Triton X-100 in 1× PBS) for 1 h, then incubated with primary antibodies for 24–48 h at 4 °C. Secondary antibodies were applied in Supermix with 200 nM/ml DAPI for 2 h at room temperature (NEUN: donkey anti-mouse 647, Invitrogen A31571, 1:200; GFAP: donkey anti-rabbit 647, Invitrogen A31573, 1:200). Sections were washed three times with 1× PBST (0.1% Tween-20 in 1× PBS), embedded in Anti-Fade Fluorescence Mounting Medium (Abcam), and covered with glass slides (Menzel-Gläser). Slides were dried at 4 °C and stored at this temperature until imaging. Primary antibodies included NEUN (Millipore, MAB377) and GFAP (Cell Signaling Technology, D1F4Q).

Images of RNAscope-treated sections were acquired at 20× magnification using an Axio Imager.Z2 microscope (Zeiss, Germany) and Zeiss ZEN 3.3 (blue edition) software. Whole-section images were collected as tiled scans in the following channels: DAPI (50 ms), FITC (200 ms for *Slc17a6* and *Slc32a1*; 500 ms for NEUN and GFAP), and Cy5 (500 ms). Image processing for figure representation was performed in ZEN 3.3, with targeted brain structures and outline of brain areas and nuclei identified using the Allen Mouse Brain Atlas (mouse.brain-map.org/experiment/thumbnails/100048576?image_type=atlas). Outlines in figures are approximations for guidance. All images were converted to colour-blindness-friendly pseudo-colours using ZEN 3.3 software.

### Data handling and statistics

*qRT-PCR*: Melting curves were compared with negative controls to confirm amplification of a single product. Normalized and relative mRNA expression of *Slc6a5* and *Slc6a9* was calculated using the delta Ct method for multiple reference genes [53], with primer efficiency differences accounted for. Replicates requiring > 45 cycles for amplification and biological outliers were manually excluded prior to data visualization. Outliers were identified using the Grubbs test (α = 0.05) in GraphPad Prism version 10.0.4. Outliers are specified in the figure legends.

Log₂ fold differences were calculated using *Actb* as the calibrator and presented in combined scatter-bar plots (mean log₂ difference relative to *Actb*). Normality of qRT-PCR values for female and male mice was assessed using the Shapiro–Wilk test. Expressional differences relative to negative controls were evaluated using the Kruskal–Wallis and Mann–Whitney U tests. Sex-dependent differences for each tissue and primer were analysed using two-tailed Mann–Whitney U or unpaired t-tests, with and without outliers. Statistical significance was set at *p* < 0.05. All analyses were performed in GraphPad Prism version 10.0.4.

*RNAscope*: Images were processed for figure representation using Zeiss ZEN 3.3 (blue edition). Targeted brain structures were identified and outlined with reference to the Allen Mouse Brain Atlas (https://mouse.brain-map.org/experiment/thumbnails/100048576?image_type=atlas). All images were converted to colour-blindness-friendly pseudo-colours in ZEN 3.3. Quantification was performed using the Fiji Cell Counter plug-in [54], criterion for positive cells was ≥ 5 dots within the same cell.

## Results

### Slc6a9 is broadly expressed in both excitatory and inhibitory neurons in the nervous system, while Slc6a5 displays a more limited distribution

**Fig. 1:**
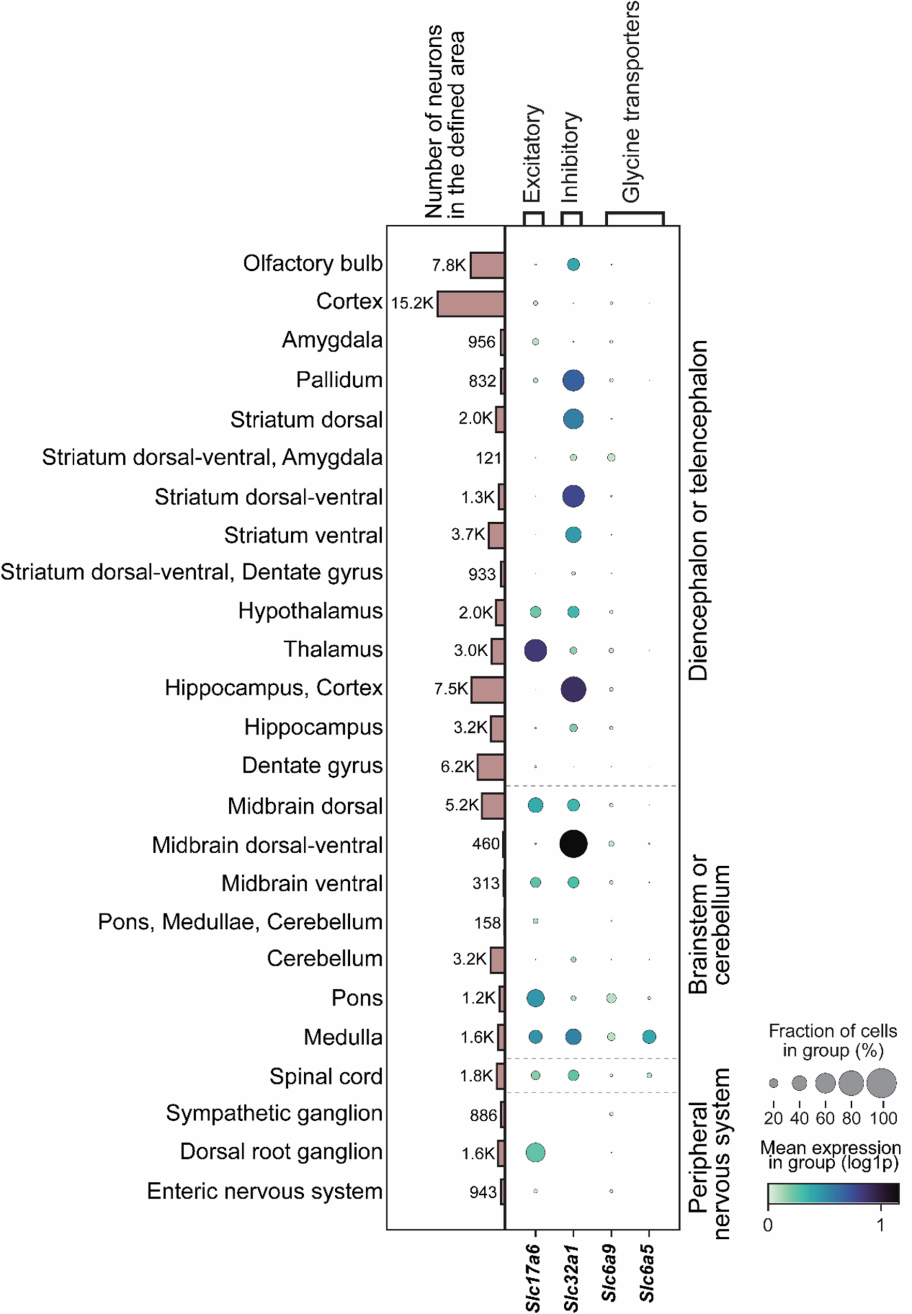
*Slc6a9 is broadly expressed in both excitatory and inhibitory neurons in the nervous system, while* *Slc6a5* *displays a more limited distribution*. *Slc6a9 *and *Slc6a5* expression was analysed in relation to the reference markers *Slc17a6* (vesicular glutamate transporter) and *Slc32a1* (vesicular inhibitory amino acid transporter) across defined regions of the central and peripheral nervous system in the Zeisel et al. (2018) dataset [42]. The dot plot illustrates expression patterns of the targeted genes in neurons within the annotated regions. Both *Slc6a9* and *Slc6a5* showed lower occurrence relative to *Slc17a6* and *Slc32a1*. *Slc6a9* was detected in all examined regions with varying prevalence, while *Slc6a5*-positive neurons were limited to the cortex, pallidum, thalamus, dentate gyrus, midbrain, cerebellum, pons, medulla, and spinal cord. Expression was defined as log1p > 0.1

**Fig. 2:**
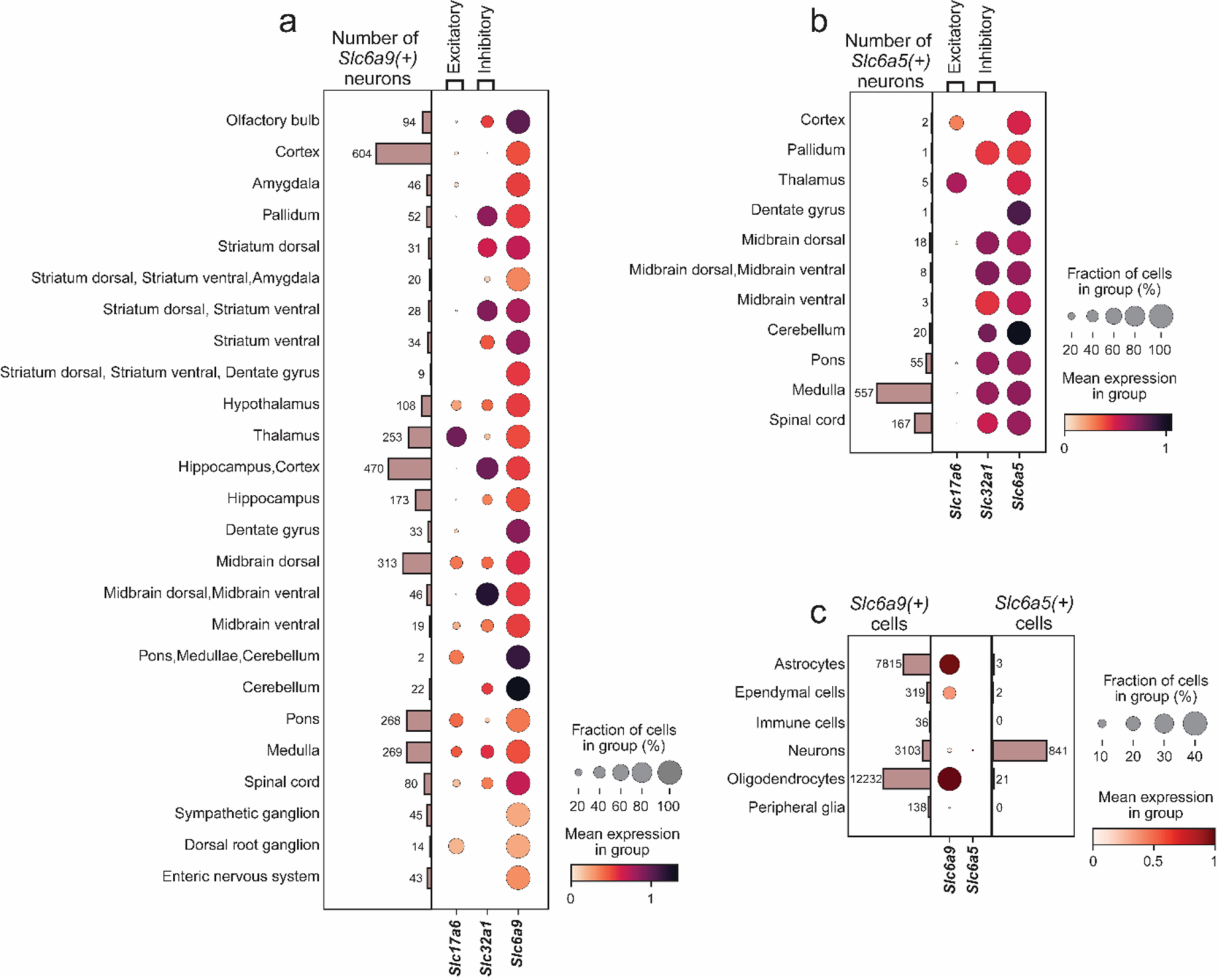
*Slc6a9-expressing neurons exhibit both excitatory and inhibitory molecular characteristics across several nervous system regions, whereas Slc6a5-expressing neurons are primarily inhibitory.* Dot plots illustrate the occurrence of *Slc6a9*-expressing neurons and *Slc6a5*-expressing neurons, along with their co-expression with *Slc17a6* and *Slc32a1* across nervous system regions. (**a**) *Slc6a9* was detected in both excitatory and inhibitory neurons, with some regions showing exclusively excitatory or inhibitory *Slc6a9* neurons, while others included both types. (**b**) *Slc6a5* occurred in excitatory and inhibitory neurons; however, in all regions except the cortex and thalamus, the majority of *Slc6a5*-positive cells co-expressed *Slc32a1*. **c**) Dot plots illustrate the occurrence of *Slc6a9* and *Slc6a5* in astrocytes, ependymal cells, CNS immune cells, neurons, oligodendrocytes and peripheral glia. Expression was defined as log1p > 0.1

Both *Slc6a9* and *Slc6a5* exhibited lower expression than the reference markers *Slc17a6* (excitatory neurons) and *Slc32a1* (inhibitory neurons) (Figs. 1 and 2a and b). While GLYT1 has traditionally been considered glial [55], re-analysis of the Zeisel et al. (2018) dataset [42] identified *Slc6a9*-expressing neurons throughout the nervous system (Fig. 1), as well as in glial cells (Fig. 2c). Occurrences exceeding 10% were observed in the dorsal–ventral striatum, amygdala, dorsal–ventral midbrain, medulla, and pons (Fig. 1; Table 1), while *Slc6a9* expression was present across all regions (Table 1). *Slc6a5* expression was comparatively low, with occurrence exceeding 1% in the dorsal–ventral midbrain, cerebellum, pons, medulla, and spinal cord (Table 1). Notably, most regions annotated in the Zeisel et al. (2018) dataset [42] did not express *Slc6a5* (Fig. 1; Table 1).

**Table 1:** Regional occurrence of *Slc6a9* and *Slc6a5* expression across nervous system regions annotated in the single-cell RNA sequencing dataset from Zeisel et al. (2018) [42]. Genes were considered expressed if log1p > 0.1. ND = not detected; NaN = not a number

Nervous system area	Total number of cells	Number of *Slc6a9*- expressing cells	Relative abundance of *Slc6a9* expression in area (%)	Number of *Slc6a5*- expressing cells	Relative abundance of *Slc6a5* expression in area (%)
Olfactory bulb	7763	94	1.21	ND	NaN
Cortex	15,205	604	3.97	2	0.01
Amygdala	956	46	4.81	ND	NaN
Pallidum	832	52	6.25	1	0.12
Striatum dorsal	1962	31	1.58	ND	NaN
Striatum dorsal–ventral, Amygdala	121	20	16.53	ND	NaN
Striatum dorsal–ventral	1332	28	2.10	ND	NaN
Striatum ventral	3676	34	0.93	ND	NaN
Striatum dorsal–ventral, Dentate gyrus	933	9	0.97	ND	NaN
Hypothalamus	1981	108	5.45	ND	NaN
Thalamus	3029	253	8.35	5	0.17
Hippocampus, Cortex	7507	470	6.26	ND	NaN
Hippocampus	3210	173	5.39	ND	NaN
Dentate gyrus	6177	33	0.53	1	0.02
Midbrain dorsal	5204	313	6.02	18	0.35
Midbrain dorsal–ventral	460	46	10.00	8	1.74
Midbrain ventral	313	19	6.07	3	0.96
Pons, Medullae, Cerebellum	158	2	1.27	ND	NaN
Cerebellum	3240	22	0.68	20	0.62
Pons	1196	268	22.41	55	4.60
Medulla	1566	269	17.18	556	35.50
Spinal Cord	1790	80	4.47	167	9.33
Sympathetic ganglion	886	45	5.08	ND	NaN
Dorsal root ganglion	1580	14	0.89	ND	NaN
Enteric nervous system	943	43	4.56	ND	NaN

Interestingly, neurons expressing *Slc6a9* or *Slc6a5* co-expressed *Slc17a6* or *Slc32a1* within the same nervous system regions (Fig. 2a, b). In most areas examined, and where prevalent, both *Slc6a9* and *Slc6a5* co-localized with *Slc32a1* (Fig. 2a, b), indicating that glycine transporters are predominantly associated with inhibitory neurons, consistent with the role of *Slc32a1* in packaging glycine into synaptic vesicles. However, comparable proportions of *Slc6a9* were observed in excitatory and inhibitory neurons within the hypothalamus, midbrain, medulla, and spinal cord. Furthermore, *Slc6a9* primarily co-localized with the excitatory marker *Slc17a6* in the thalamus, pons, and dorsal root ganglion (DRG) (Fig. 2a). A similar pattern was observed for thalamic neurons in the *Slc6a5* analysis (Fig. 2b), suggesting that glycine transporter-positive neurons in this region are largely excitatory. Apart from these exceptions, *Slc6a5* was primarily co-expressed with *Slc32a1* (Fig. 2b). However, a total of 19 neurons out of 3,076 were found to co-express *Slc6a9*, *Slc17a6* and *Slc32a1* in medulla (3 neurons), pallidum (1 neuron), spinal cord (1 neuron), midbrain (7 neurons), hypothalamus (2 neurons), thalamus (4 neurons) and cortex (1 neuron), and a total of 8 neurons out of 833 were found to co-express *Slc6a5*, *Slc17a6* and *Slc32a1* in medulla.

In addition, the occurrence of both glycine transporters was examined in the sequencing dataset focusing on the different cell categories of the nervous system annotated by Zeisel et al. (2018) [42]. *Slc6a9* mRNA expression was found to occur in astrocytes (32%), ependymal cells (17%), oligodendrocytes (39%), central nervous system immune cells (i.e., microglia, 0.5%) and peripheral glia (1.2%), while the same occurrence in neurons was 4% (Fig. 2c, Additional file 1: Fig. S1a). Interestingly, *Slc6a5* was found exclusively in neurons (1%), while the occurrence in glial cells was 0.1% or lower (ependymal cells 0.1%, oligodendrocytes 0.06%, astrocytes 0.01%, peripheral glia and central nervous system immune cells 0%) (Additional file 1: Fig S1b). To further validate the expression in glial cells, the co-occurrence of *Slc6a9* or *Slc6a5* with *Gfap* (astrocytic marker [56, 57]), *Mbp* (oligodendrocyte marker [58, 59]) and *Foxj1* (ependymal cell marker [60, 61]) was analysed in the cerebellum and spinal cord (Additional file 1: Fig. S1c, d). These two regions were chosen because of a high occurrence of glial cells and the criterion of the code was to plot brain regions with more than five glial cells expressing one of the glycine transporters. *Slc6a9* was found to primarily co-express *Mbp* and *Gfap* in the cerebellum (Additional file 1: Fig. S1c), while in the spinal cord, *Slc6a9* was found to primarily co-express *Foxj1* and *Mbp* (Additional file 1: Fig S1d). On the other hand, *Slc6a5* was found to have minimum overlap with the glial markers in both the cerebellum and spinal cord (Additional file 1: Fig. S1c, d).

Taken together, these findings demonstrate that *Slc6a9* and *Slc6a5* are expressed in central nervous system neurons, with *Slc6a9* being more prevalent throughout the nervous system areas, as well as in glial cells. Notably, both transporters are present in subsets of excitatory and inhibitory neurons.

### Sex-dependent differences in mRNA expression of Slc6a9 and Slc6a5 were detected using qRT-PCR

Re-analysis of the single-cell dataset revealed abundant *Slc6a9* expression and a more restricted *Slc6a5* expression in the central and peripheral nervous system. The Zeisel et al. (2018) dataset [42], however, comprises pooled neurons from female and male mice, preventing the identification of sex-dependent differences. To address this, bulk tissue samples were collected from both female and male mice, and analysed by qRT-PCR to evaluate sex-dependent variation in gene expression.

**Fig. 3:**
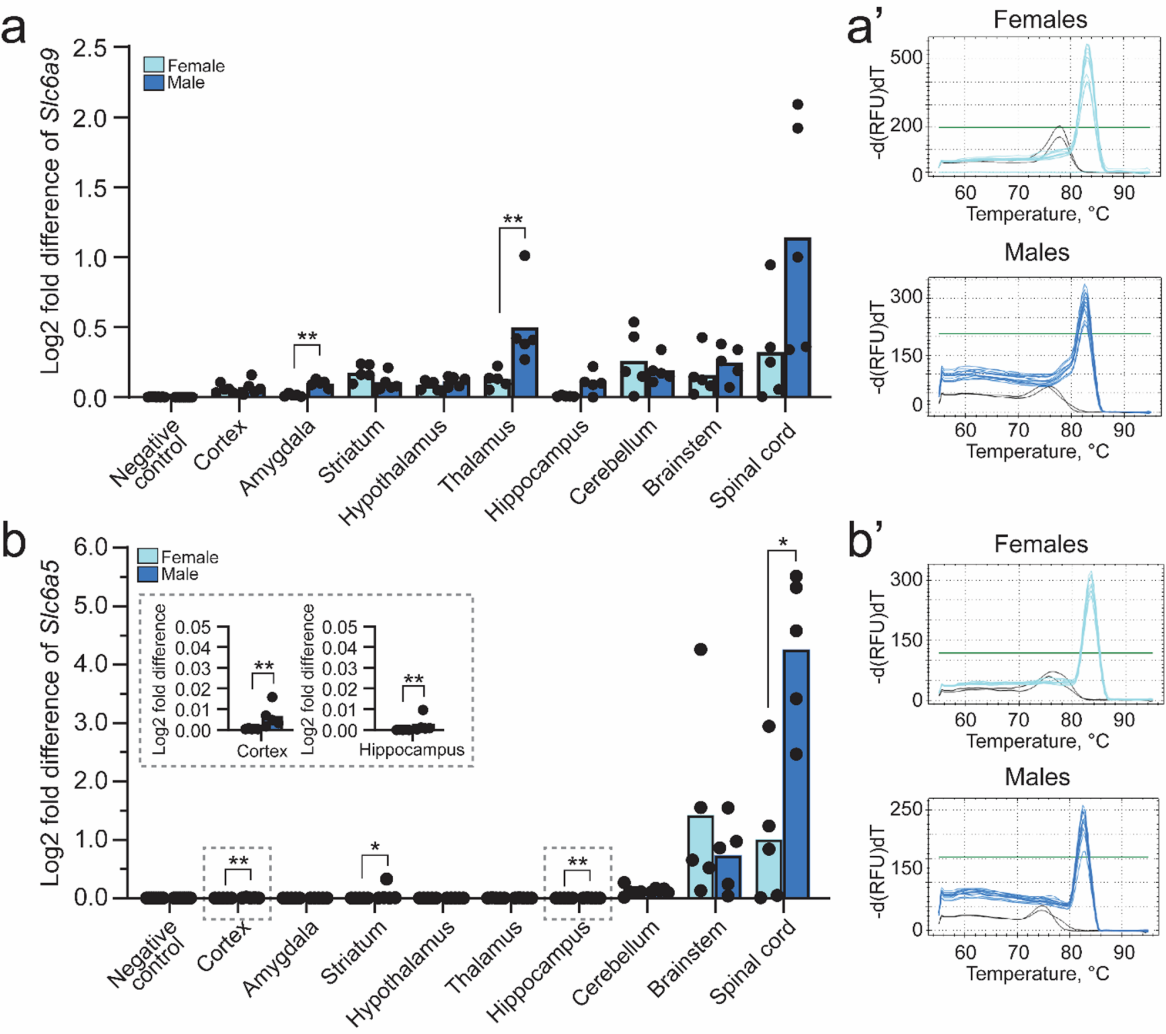
*Sex-dependent differences in mRNA expression of Slc6a9 and Slc6a5 could be detected using qRT-PCR.* Expression of *Slc6a9* and *Slc6a5* in adult female (n = 5) and adult male (n = 5) C57BL/6J mice were observed using qRT-PCR, with a cut-off of 45 cycles. Relative mRNA expression was calculated using the delta Ct method with three stable reference genes (*Cyclo*, *Rpl19*, *Gapdh*) and *Actb* was used as the calibrator. Stable reference genes were identified using the GeNorm protocol [53]. No biological outliers were identified for nervous system tissue samples using the Grubbs outlier test with α = 0.05 prior to analysis. The Log_2_ fold mean difference, with individual dots representing individual mice relative to *Actb* expression, is illustrated in the combined scatter-bar plot. Normality was assessed using the Shapiro-Wilk test. Difference between female and male mice for each tissue were calculated using a two-tailed Mann–Whitney U-test, where *p < 0.05, **p < 0.01. (**a**) *Slc6a9* expression in central nervous system tissues of females and males, with differences in each region as follows: cortex (p = 0.6905), amygdala (p = 0.0079), striatum (p = 0.0952), hypothalamus (p = 0.2222), thalamus (p = 0.0079), hippocampus (p = 0.1508), cerebellum (p = 0.8413), brainstem (p = 0.4206), and spinal cord (p = 0.0556). (**b**) *Slc6a5* expression in central nervous system tissues of females and males, with differences in each regions investigated. An enlargement with appropriate y-axis scaling is shown for the cortex and the hippocampus in the dashed box: cortex (p = 0.0079), amygdala (p = 0.1508), striatum (p = 0.0317), hypothalamus (p = 0.5476), thalamus (p = 0.1508), hippocampus (p = 0.0079), cerebellum (p = 0.6905), brainstem (p = 0.6905), and spinal cord (p = 0.0159). Representative melt peak images for females (light blue), males (blue) and negative controls (black) are shown in panels a’–b’

Overall, *Slc6a9* and *Slc6a5* showed low expression in bulk tissue samples from the nervous system and peripheral organs (Fig. 3, Additional file 1: Fig. S2). However, all amplified samples exhibited a single melting peak with identical melting temperatures (Fig. 3a’–d’), confirming the reliability of the qRT-PCR results. To assess gene expression in specific tissues, comparisons were made with negative controls (Additional file 1: Table S1). For *Slc6a9*, expression was detectable above background levels in the striatum, hypothalamus, thalamus, cerebellum, brainstem, and spinal cord of female mice, whereas in male mice it was observed only in the thalamus, cerebellum, brainstem, and spinal cord. This pattern suggests a more caudal distribution compared with the single-cell analysis (Fig. 1). Consistent with the single-cell data, *Slc6a5* expression was detectable above background levels in the brainstem and spinal cord of females, and in the striatum, cerebellum, brainstem, and spinal cord of males. Regarding visceral organs, *Slc6a9* expression was observed in the thymus, lung, and spleen of female mice, but restricted to the liver in males (Additional file 1: Fig. S2). By contrast, *Slc6a5* expression was not detectable above background levels in any of the internal organs examined (Additional file 1: Table S1).

When normalized relative Ct values were compared across central nervous system regions, sex-dependent differences were observed for *Slc6a9* in the amygdala (*p* = 0.0079) and thalamus (*p* = 0.0079) (Fig. 3a), and for *Slc6a5* in the cortex (*p* = 0.0079), hippocampus (*p* = 0.0079), striatum (*p* = 0.0317), and spinal cord (*p* = 0.0159) (Fig. 3b), with males consistently exhibiting higher expression levels than females. When comparing the normalized relative Ct values of *Slc6a9* and *Slc6a5* in relation to *Actb*, the analyses revealed that *Slc6a5* expression in the spinal cord was nearly fourfold higher than *Slc6a9* in males, but only twofold higher in females (Fig. 3a, b). In contrast, higher *Slc6a5* expression relative to *Slc6a9* was observed in the brainstem of females, suggesting sex-dependent differences in glycine transporter expression patterns in caudal brain regions.

In peripheral organs, higher *Slc6a9* expression was observed in males in liver (*p* = 0.0079 without outlier; *p* = 0.0119 with outlier), intestine (*p* = 0.0159 without outlier; *p* = 0.0079 with outlier), spleen (*p* = 0.0179 without outlier; *p* = 0.0117 with outlier), and kidney (*p* = 0.0079 with and without outlier), whereas lower expression was detected in genitalia compared with females (Additional file 1: Fig. S2a). No sex-dependent differences in *Slc6a9* were observed in the thymus or lung.

Although *Slc6a5* expression could not be distinguished from negative controls in internal organs, sex-dependent differences were still detected in the thymus (*p* = 0.0952 without outlier; *p* = 0.0079 with outlier), lung (*p* = 0.0357 without outlier; *p* = 0.0079 with outlier), and intestine (*p* = 0.0159 without outlier; *p* = 0.0079 with outlier) (Additional file 1: Fig. S2b).

Taken together, qRT-PCR analyses revealed sex-dependent differences in the expression of the glycine transporters *Slc6a9* and *Slc6a5*. In the central nervous system, higher expression was generally observed in males, with *Slc6a9* differences detected in the amygdala and thalamus, and *Slc6a5* differences in the cortex, hippocampus, striatum, and spinal cord. *Slc6a5* expression was found to be markedly higher than *Slc6a9* in the spinal cord, whereas relatively higher *Slc6a5* expression was observed in the brainstem of females. In peripheral organs, *Slc6a9* was broadly expressed and exhibited sex-dependent variation, whereas *Slc6a5* expression was largely undetectable but still showed measurable sex differences in the thymus, lung, and intestine. Overall, *Slc6a9* was more widely distributed than *Slc6a5*, and both transporters displayed distinct sex-dependent expression patterns.

### Histological analysis revealed that Slc6a9 is distributed throughout the central nervous system, predominantly in glial fibrillary acidic protein (GFAP)-positive cells

**Fig. 4:**
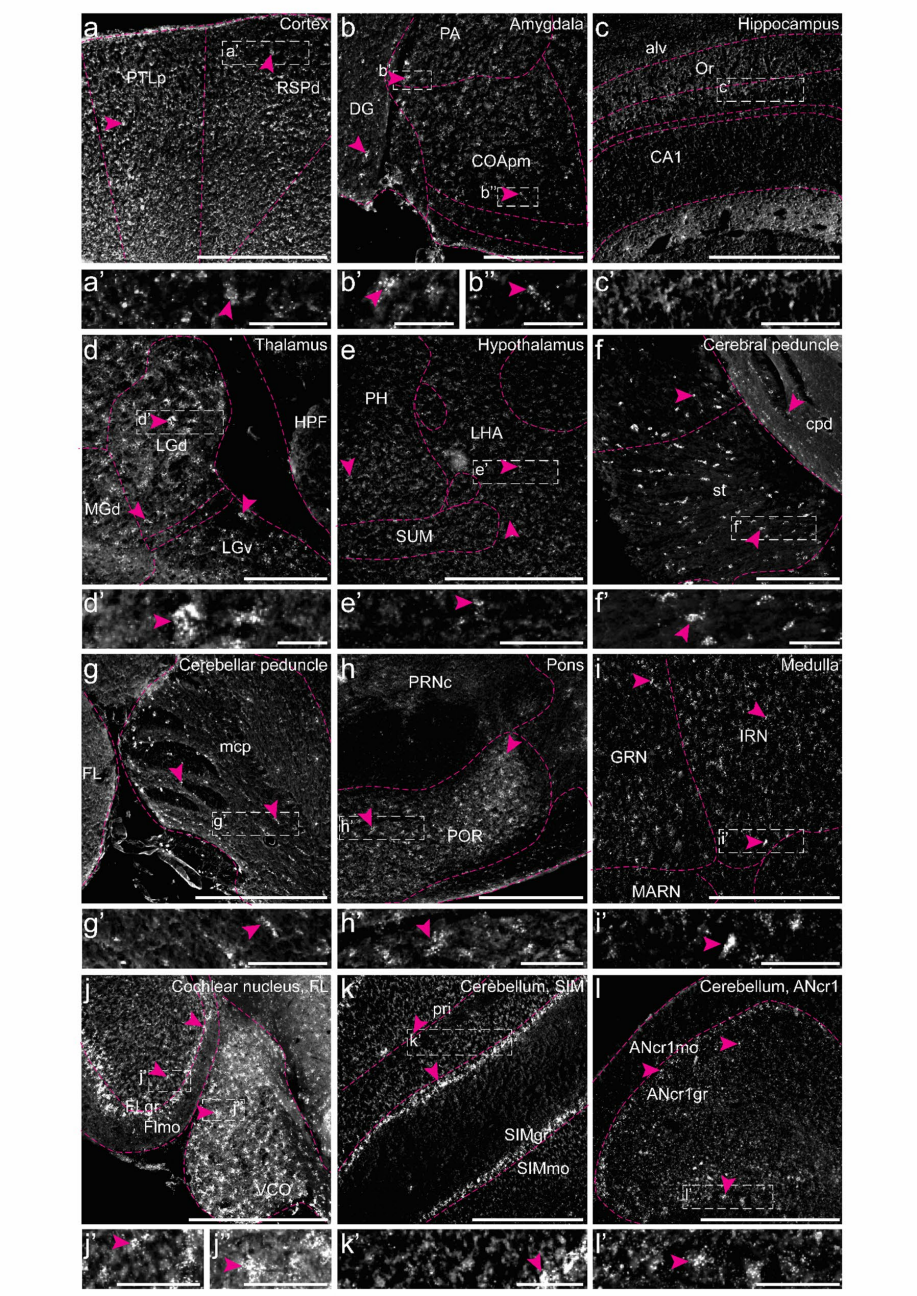
*Histological analysis of Slc6a9 expression in the adult mouse brain using RNAscope.* Expression was examined in the (**a**) cortex, (**b**) amygdala, (**c)** hippocampus, (**d**) thalamus, (**e**) hypothalamus, (**f**) cerebral peduncle, (**g**) cerebellar peduncle, (**h**) pons, (**i**) medulla, (**j**) cochlear nucleus, and (**k**–**l**) cerebellum. Representative images are shown from one male, with Bregma coordinates (**a**, **c**) − 2.18 mm, (**b**, **d**–**f**) − 2.92 mm, (**g**, **h**) − 4.24 mm, and (**i**–**l**) − 6.24 mm, enlargements in a’–l’ marked by dashed boxes. *Slc6a9* signals are pseudo-coloured in light grey, and magenta arrows indicate examples of representative cells with expression (≥ 5 dots within the same cell). Scale bars: a–l 500 μm, enlargements 100 μm. Abbreviations: alv = alveus; ANcr1gr = Ansiform lobule crus 1, granular layer; ANcr1mo = Ansiform lobule crus 1, molecular layer; CA1 = Cornu Ammonis 1; COApm = cortical amygdalar area, posterior part, medial zone; cpd = cerebral peduncle; DG = dentate gyrus; FL = flocculus; FLgr = flocculus, granular layer; FLmo = flocculus, molecular layer; GRN = gigantocellular reticular nucleus; HPF = Hippocampal formation; IRN = intermediate reticular nucleus; LGd = dorsal lateral geniculate body; LGv = ventral lateral geniculate body; LHA = lateral hypothalamic area; MARN = magnocellular reticular nucleus; mcp = middle cerebellar peduncle; MGd = dorsal nucleus of medial geniculate body; Or = oriens layer of the hippocampus; PA = posterior amygdalar nucleus; PH = posterior hypothalamic nucleus; POR = periolivary region; pri = Purkinje cell layer; PRNc = pontine reticular nucleus; PTLp = posterior parietal association areas; RSPd = retrosplenial area, dorsal part; SIMgr = simple lobule, granular layer; SIMmo = simple lobule, molecular layer; st = stria terminalis; SUM = supramammillary nucleus; VCO = ventral cochlear nucleus

*Slc6a9* has primarily been reported as expressed in glial cells located near glycinergic neurons [55, 62] and is known to be present in the cortex, hippocampus, brainstem, and spinal cord [55, 63]. However, re-analysis using scRNA-seq and qRT-PCR revealed that mRNA expression of this transporter occurs throughout the central nervous system, prompting further investigation of its spatial distribution. RNAscope confirmed that *Slc6a9* was expressed in multiple brain regions in mice (Fig. 4). Specifically, *Slc6a9*-positive cells were detected in the grey matter of the cortex, amygdala, thalamus, hypothalamus, brainstem, and cerebellum (Fig. 4a–l) in both female and male mice. No expression was observed in the Cornu Ammonis (CA) of the hippocampus (Fig. 4f); however, *Slc6a9* was detected in the dentate gyrus (Fig. 4g). In addition, *Slc6a9*-expressing cells were present in the cerebral and cerebellar peduncles (Fig. 4f, g).

**Fig. 5:**
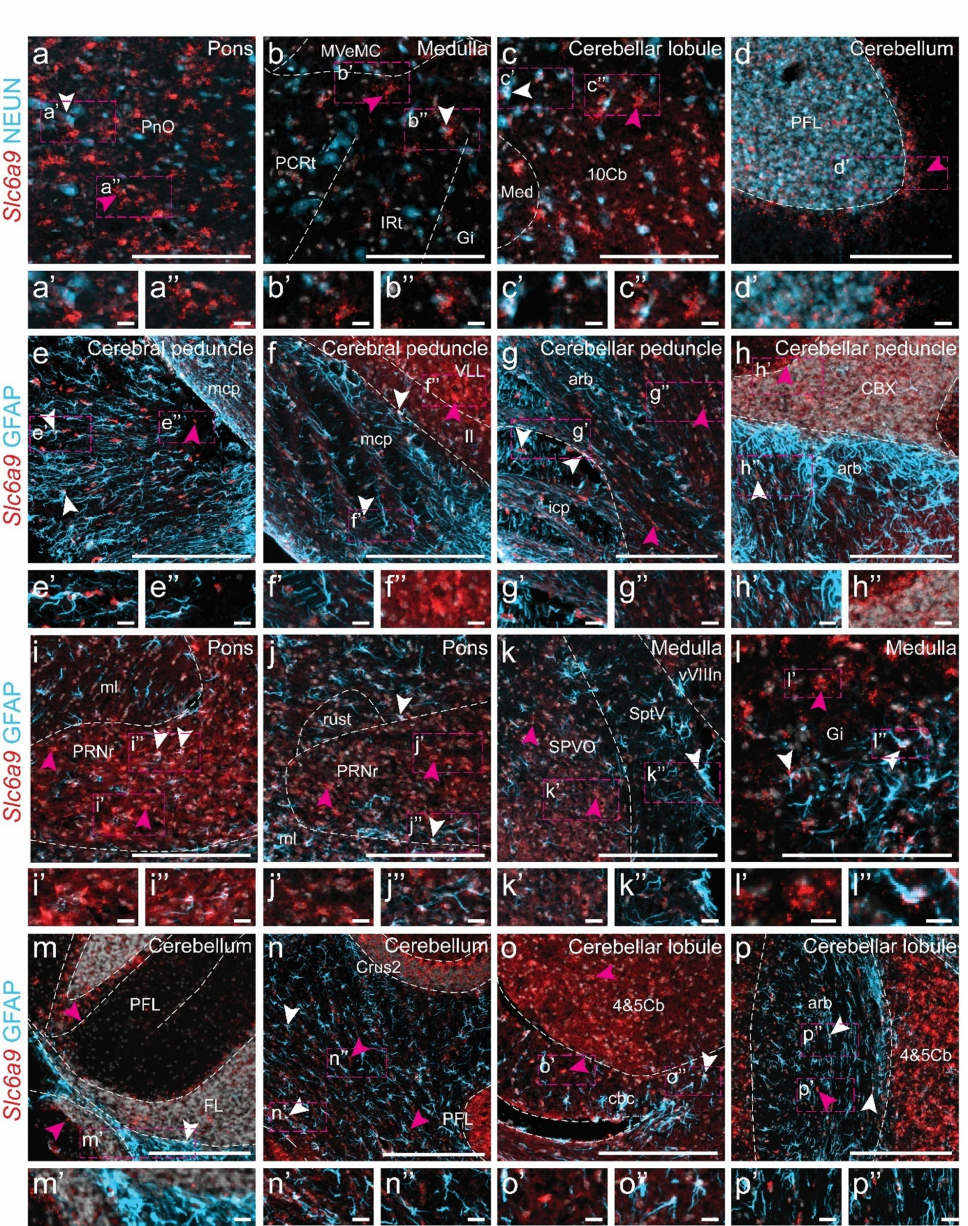
In situ *hybridization and immunohistochemistry analysis of Slc6a9 co-expression with neuronal and astrocytic markers in the adult mouse brain.* Fluorescent RNAscope for *Slc6a9 *(red) was combined with immunohistochemistry for NEUN (neuronal marker, light blue) or GFAP (astrocytic marker, light blue). Nuclei were stained with DAPI (light grey). Sections at Bregma (**a**, **e**, **f**, **i**, **j**) − 4.24 mm and (**b**–**d**, **g**, **h**, **k**–**p**) − 6.24 mm were used to assess co-expression with NEUN in the (**a**) pons, (**b**) medulla oblongata, (**c**) cerebellar lobule, and (**d**) paraflocculus (PFL) of cerebellum. Sections at the same Bregma levels were used to assess co-expression with GFAP in the cerebral peduncles of (**e**) female and (**f**) male mice, cerebellar peduncles of (**g**) female and (**h**) male mice, pons of (**i**) female and (**j**) male mice, medulla oblongata of (**k**) female and (**l**) male mice, paraflocculus/flocculus (FL)/crus 2 of (**m**) female and (**n**) male mice, and cerebellar lobule of (**o**) female and (**p**) male mice. Representative images are shown from two males, with similar expression observed in females, enlargements in a’–p’’ marked by dashed boxes. White arrows indicate examples of co-expressing cells, while magenta arrows highlight examples of cells expressing *Slc6a9* only (≥ 5 dots within the same cell). Scale bar: a–p 100 μm, enlargements 20 μm. Abbreviations: 4&5Cb = fourth and fifth cerebellar lobules; 10Cb = tenth cerebellar lobule; arb = arbor vitae; cbc = cerebellar commissure; CBX = cerebellar cortex; Crus2 = crus 2 of the ansiform lobule; FL = flocculus; Gi = gigantocellular reticular nucleus; icp = inferior cerebellar peduncle; IRt = intermediate reticular nucleus; ll = lateral lemniscus; mcp = middle cerebellar peduncle; Med = medial (fastigial) cerebellar nucleus; ml = medial lemniscus; MVeMC = medial vestibular nucleus, magnocellular part; PCRt = parvicellular reticular nucleus; PFL = paraflocculus; PnO = pontine reticular nucleus, oral part; PRNr = pontine reticular nuclues; rust = rubrospinal tract; SptV = spinal tract of the trigeminal nerve; SPVO = spinal nucleus of the trigeminal, oral part; VLL = ventral nucleus of the lateral lemniscus; vVIIIn = vestibular nerve. Separate channels can be found in Additional file 1: Fig. S3

Previous reports suggest that *Slc6a9* is primarily located to glial cells, although expression has also been noted in certain neuronal populations [55, 64, 65]. To further investigate this, RNAscope was combined with immunohistochemistry using the neuronal marker NEUN and the astrocytic marker GFAP. *Slc6a9* was detected in both NEUN- and GFAP-positive cells, as well as in NEUN- and GFAP-negative cells (Fig. 5), predominantly within the grey matter. This pattern indicates that *Slc6a9* is expressed in both neurons and astrocytes, and may be expressed in neuronal populations not marked by NEUN or in other glial cell types (Fig. 5). Expression patterns were qualitatively similar in females and males (Fig. 5e–p), although differences in signal intensity between sections (Figs. 4 and 5) were observed, likely reflecting variability in assay efficiency and tissue quality. Consequently, no quantitative analysis was performed.

**Fig. 6:**
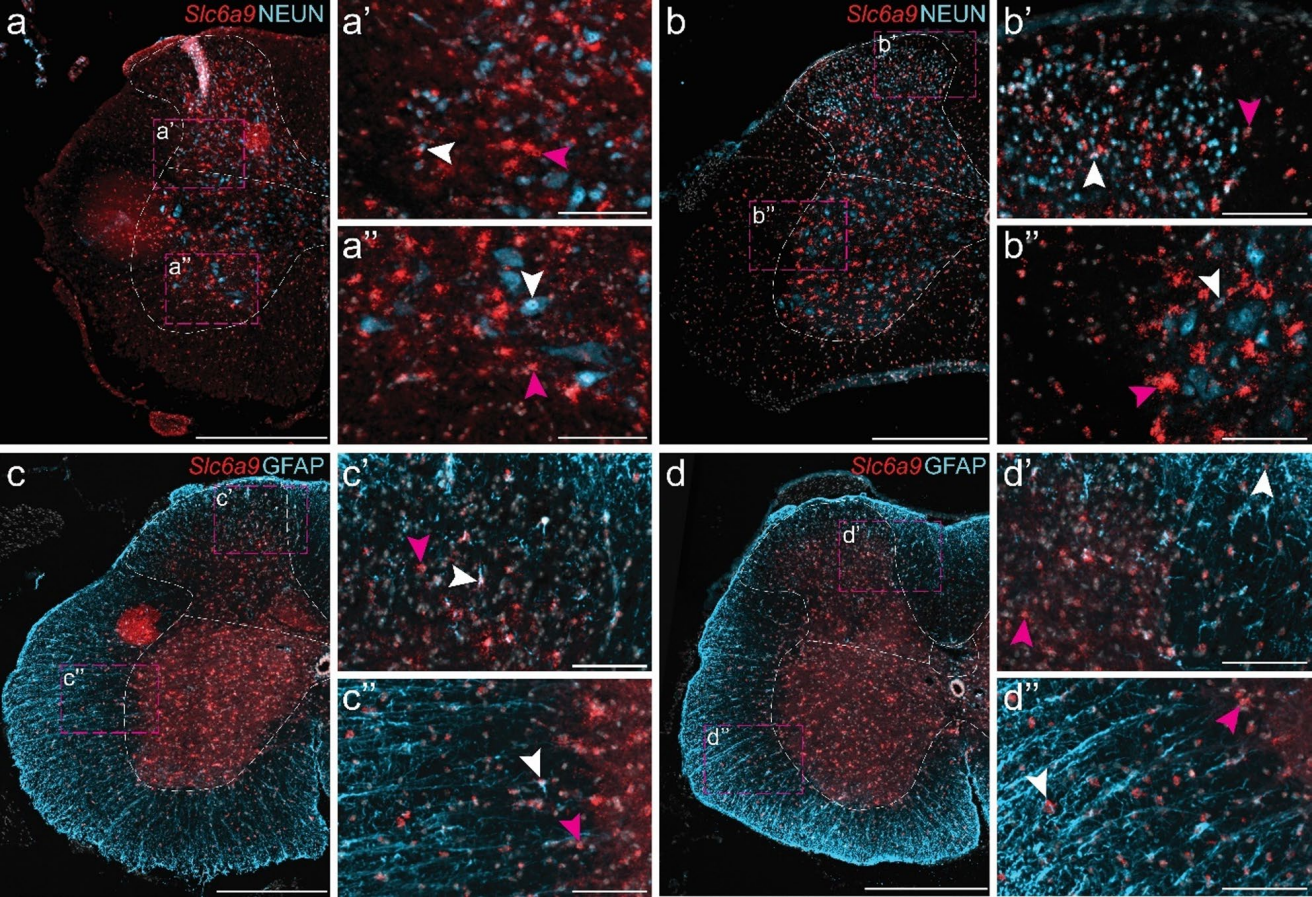
*Co-expression of Slc6a9 with neuronal and astrocytic markers in the lumbar spinal cord of adult mice.* RNAscope for *Slc6a9* (red) was combined with immunohistochemistry for NEUN (neuronal marker, light blue) and GFAP (astrocytic marker, light blue) in female and male mice. Nuclei were stained with DAPI (light grey). (**a**–**d**) Representative images from one (**a**, **c**) female and one (**b**, **d**) male show lumbar spinal cord sections between L5 (**b**, **c**) and L6 (**a**, **d**). White arrows indicate examples of double-positive cells, while magenta arrows highlight examples of cells expressing *Slc6a9* only (≥ 5 dots within the same cell). Scale bars: a–d 500 μm, enlargements 50 μm. Separate channels can be found in Additional file 1: Fig. S4

**Fig. 7:**
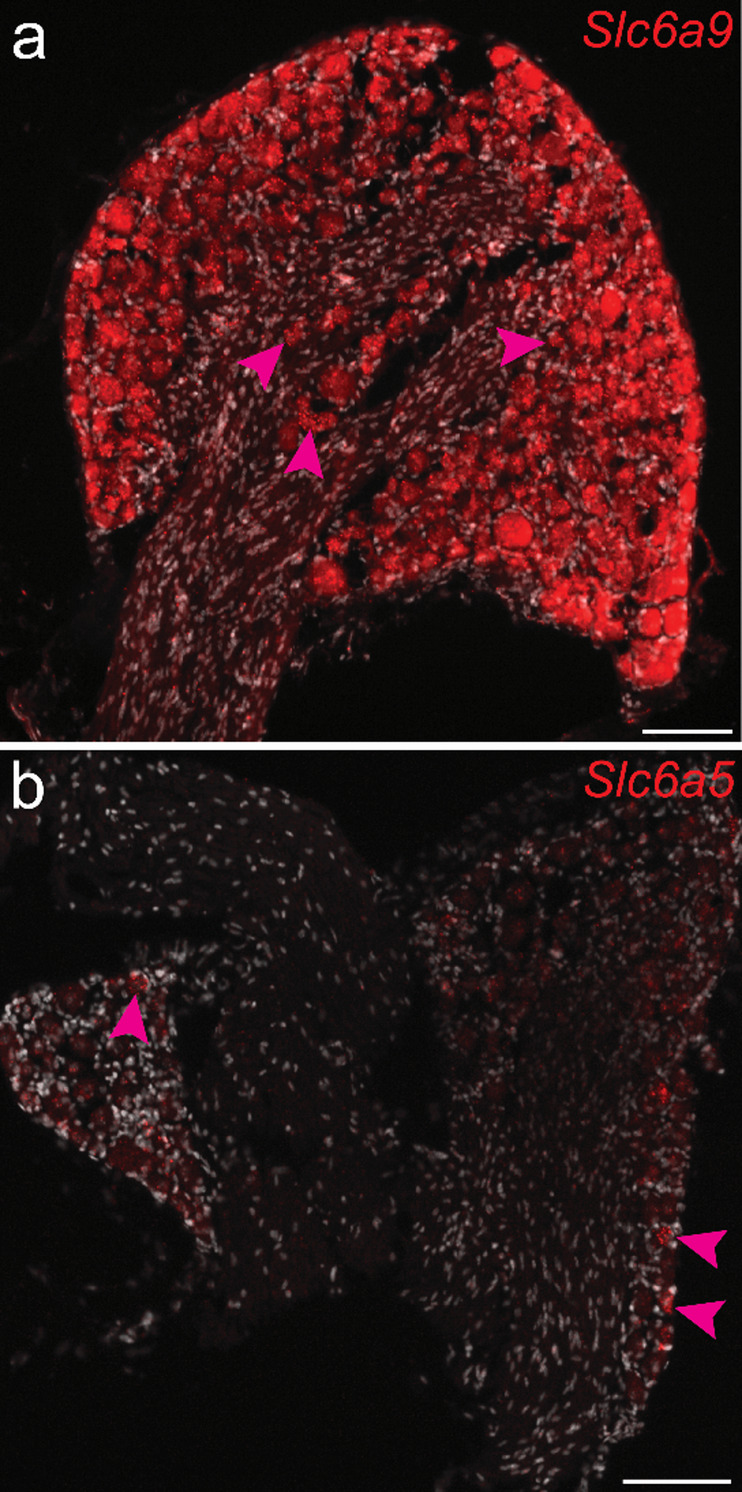
*Expression of Slc6a9 and Slc6a5 in the dorsal root ganglia (DRG) of adult mice.* RNAscope analysis in female and male mice revealed that both glycine transporters were expressed across all DRG divisions. (**a**, **b**) Representative images of (**a**) lumbar and (**b**) thoracic DRG from female mice, showing transporter signals in red and nuclei staining with DAPI (light grey). Magenta arrows indicate examples of expressing cells (≥ 5 dots within the same cell). Scale bars: 50 μm

In the spinal cord, *Slc6a9* was detected in both NEUN-positive and GFAP-positive cells (Fig. 6). While expression in NEUN-positive cells was sparse, GFAP-positive cells exhibited *Slc6a9* expression in both the grey and white matter of the dorsal and ventral horns across all spinal cord divisions. Expression was observed in both female and male mice, with no apparent sex-related differences (Fig. 6). In addition, *Slc6a9* was highly expressed in the DRG, whereas the second glycine transporter, *Slc6a5*, was detected only in a small subset of DRG cells (Fig. 7)

### Histological analysis revealed that Slc6a5 was predominantly expressed in the caudal regions of the brain and in the spinal cord, where it was mainly localized to inhibitory neurons, with limited expression observed in a subset of excitatory neurons

*Slc6a5* expression has predominantly been reported in caudal brain regions [32, 66, 67], a pattern further corroborated by the scRNA-seq and qRT-PCR data presented here (Figs. 1, 2 and 3). In contrast, a recent study employing tdTomato reporter line-based genetic labelling suggested a broader distribution of *Slc6a5* throughout the brain, although subsequent in situ analyses in male mice confirmed that expression was largely restricted to the thalamus and midbrain [67]. To refine the spatial localization of *Slc6a5*, fluorescent in situ hybridization (RNAscope) was performed. Furthermore, co-expression of *Slc32a1* and *Slc17a6* in *Slc6a5*-positive cells was assessed to determine their excitatory or inhibitory molecular characteristics.

**Fig. 8:**
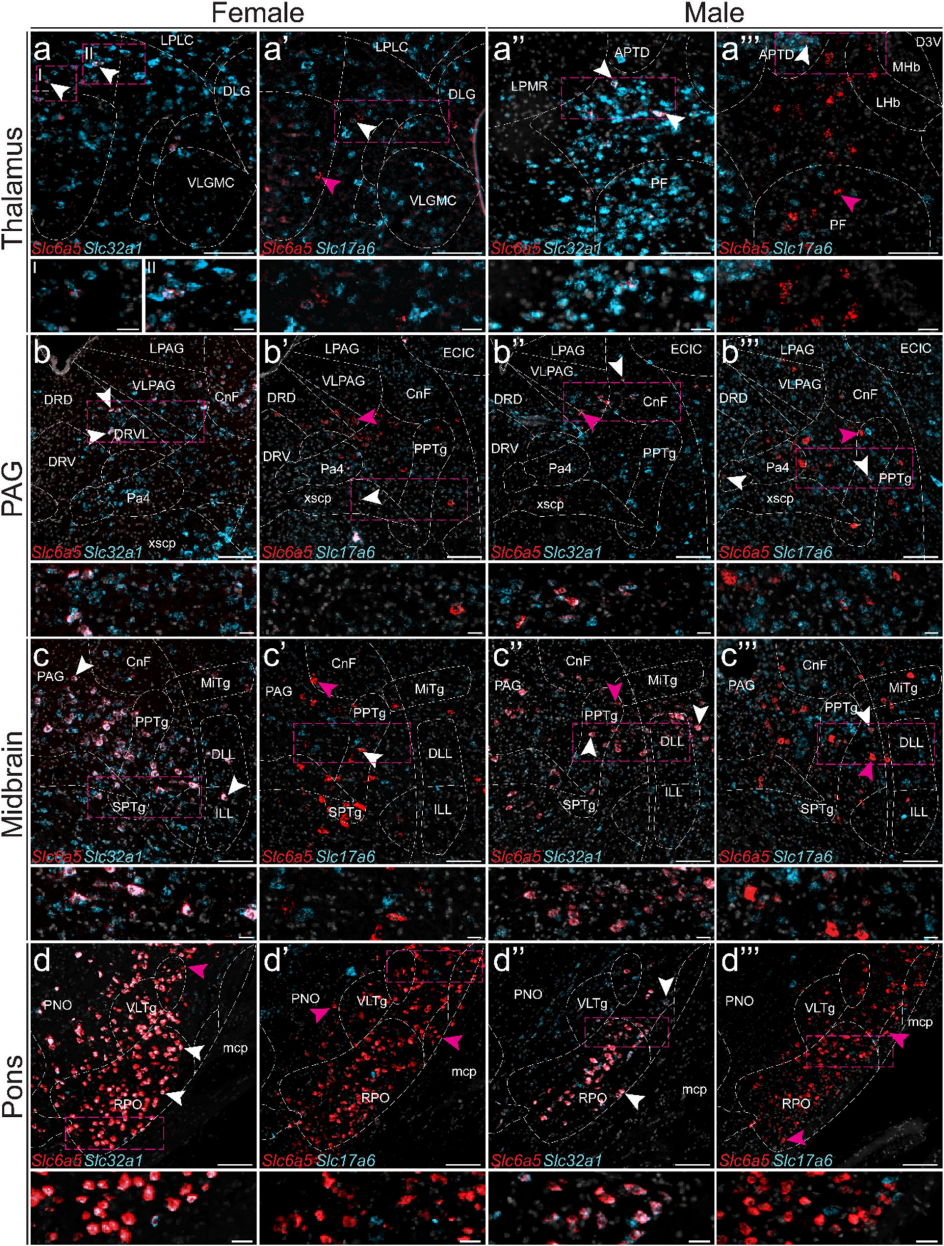
*Histological analysis of Slc6a5 expression in thalamus and midbrain*,* and its overlap with inhibitory and excitatory markers.* RNAscope was performed on sections of (**a**) thalamus, (**b**) periaqueductal grey (PAG), (**c**) midbrain, and (**d**) pons obtained from female and male mice to examine *Slc6a5* expression (red) and its co-localization with the inhibitory marker *Slc32a1* (encoding VIAAT, light blue) or the excitatory marker *Slc17a6* (encoding VGLUT2, light blue). Nuclei were stained with DAPI (light grey). Representative images (**a**–**d**’’’) show overlap with *Slc32a1* (**a**–**d**, **a**’–**d**’’) or *Slc17a6* (**a**’–**d**’, **a**’’’–**d**’’’), enlargements marked by dashed boxes. White arrows indicate examples of overlapping cells, while magenta arrows highlight examples of *Slc6a5*-expressing cells (≥ 5 dots within the same cell). Sections were collected at Bregma levels (**a**–**a**’’’) − 2.70 mm, (**b**–**c**’’’) − 4.48 mm, and (**d** – **d**’’’) − 4.72 mm. Scale bars: 100 μm, enlargements 20 μm. Abbreviations: APTD = anterior pretectal nucleus, dorsal part; CnF = cuneiform nucleus; DLG = dorsal, lateral geniculate nucleus; DLL = dorsal nucleus of the lateral lemniscus; DRD = dorsal raphe nucleus, dorsal part; DRV = dorsal raphe nucleus, ventral part; DRVL = dorsal raphe nucleus, ventrolateral part; ECIC = external cortex of the inferior colliculus; ILL = intermediate nucleus of the lateral lemniscus; LHb = lateral habenular nucleus; LPAG = lateral periaqueductal gray; LPLC = lateral posterior thalamic nucleus, laterocaudal part; LPMR = lateral posterior thalamic nucleus, mediorostral part; mcp = middle cerebellar peduncle; MHb = medial habenular nucleus; MiTg = microcellular tegmental nucleus; Pa4 = paratrochlear nucleus; PAG = periaqueductal grey; PF = parafascicular thalamic nucleus; PNO = pontine reticular nucleus, oral part; PPTg = pedunculopontine tegmental nucleus; RPO = rostral periolivary region; SPTg = subpedencular tegmental nucleus; VLGMC = ventral lateral geniculate nucleus, magnocellular part; VLPAG = ventrolateral periaqueductal grey; VLTg = ventrolateral tegmental area; xscp = decussation of the superior cerebellar peduncle. Separate channels can be found in Additional file 1: Fig. S5

A small number of *Slc6a5*-positive cells were detected in the thalamus, PAG, midbrain, and pons (Fig. 8). Within the thalamus, expression was observed around the lateral geniculate nucleus, the caudal portions of the lateral and medial posterior nuclei, and the anterior nuclei (Fig. 8a–a’’’). Additional expression was found in the ventrolateral PAG, the ventrolateral subnucleus of the dorsal raphe nucleus (Fig. 8b–b’’’), as well as in the vicinity of the cuneiform nucleus and the pedunculopontine tegmental nucleus (Fig. 8c–d’’’). These cells were present in both female and male mice, and most co-expressed the inhibitory marker *Slc32a1*, although *Slc17a6*-positive *Slc6a5* neurons were detected (Fig. 8). In the thalamus, 74 ± 1% (female – range of analysed sects.  70–83%, *n* = 2 mice, 2 sections per mouse) and 77 ± 22% (male – range of analysed sects.  50–88%, *n* = 1 mouse, 3 sections) of the *Slc6a5* labelled cells co-expressed *Slc32a1*, while only 15 ± 6% (female – range of analysed sects.  8–19%, *n* = 1 mouse, 3 sections) and 9 ± 4% (male – range of analysed sects.  4–100%, *n* = 2 mice, 4–5 section per mouse) of the *Slc6a5* labelled cells co-expressed *Slc17a6*. In the PAG region, a majority of the *Slc6a5* labelled cells co-expressed *Slc32a1* (female – 88 ± 7%, range of analysed sects.  80–93%, *n* = 2 mouse, 3 sections per mouse; male – 87 ± 7%, range of analysed sects.  67–98%, *n* = 2 mice, 1–6 section per mouse); however, a proportion of the *Slc6a5* labelled cells co-expressed *Slc17a6* (female – 4 ± 2%, range of analysed sects.  0–12%, *n* = 2 mice, 2 sections per mouse; male – 7 ± 1%, range of analysed sects.  0–15%, *n* = 2 mice, 1–5 sections per mouse). Similar findings were observed in the midbrain (female – *Slc6a5/Slc32a1* proportion: 74 ± 8%, range of analysed sects.  27–97%, *n* = 2 mice, sects.  2–4 per mouse; *Slc6a5/Slc17a6* proportion: 2 ± 1%, range of analysed sects.  0–7%, *n* = 2 mice, 1–3 sections per mouse; male – *Slc6a5/Slc32a1* proportion: 87 ± 7%, range of analysed sects.  60–95%, *n* = 2 mice, 1–10 sections per mouse); *Slc6a5/Slc17a6* proportion: 5 ± 1% (range of analysed sects.  0–15%, *n* = 2 mice, 1–10 sections per mouse) and pons (female – *Slc6a5/Slc32a1* proportion: 97 ± 2%, range of analysed sects.  91–100%, *n* = 2 mice, sects.  2–4 per mouse; *Slc6a5/Slc17a6* proportion: 2 ± 1%, range of analysed sects.  1–3%, *n* = 1 mice, 3 sections per mouse; male – *Slc6a5/Slc32a1* proportion: 86 ± 8%, range of analysed sects.  64–100%, *n* = 2 mice, 1–4 sections per mouse; *Slc6a5/Slc17a6* proportion: 5 ± 1%, range of analysed sects.  2–11%, *n* = 2 mice, 1–7 sections per mouse) of female and male mice.

**Fig. 9:**
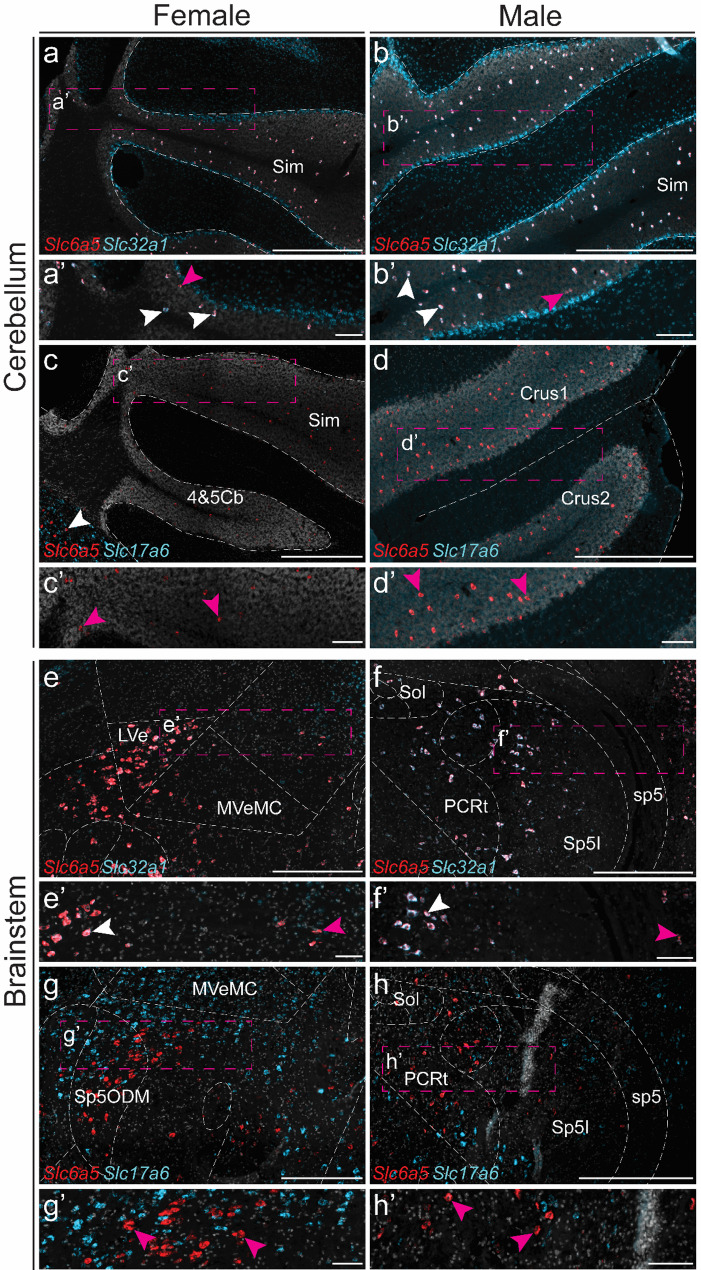
*Slc6a5 expression analysis reveals a predominantly inhibitory subpopulation of cells in the cerebellum and brainstem.* Fluorescent in situ hybridization was performed on sections at Bregma (**e**–**h**’) − 5.88 and (**a**–**d**’) − 6.64 mm from female and male mice to examine *Slc6a5* expression (red) and its co-expression with inhibitory (*Slc32a1*, encoding VIAAT, light blue) or excitatory (*Slc17a6*, encoding VGLUT2, light blue) markers in the (**a**–**d**’) cerebellum and (**e**–**h**’) brainstem. Nuclei were stained with DAPI (light grey). Abbreviation: 4&5Cb = fourth and fifth cerebellar lobules; Crus1 = crus 1 of the ansiform lobule; Crus2 = crus 2 of the ansiform lobule; LVe = lateral vestibular nucleus; MVeMC = medial vestibular nucleus, magnocellular part; PCRt = parvicellular reticular nucleus; Sim = simple lobule; Sol = solitary tract; sp5 = spinal trigeminal tract; Sp5I = spinal trigeminal nucleus, interpolar part; Sp5ODM = spinal trigeminal nucleus, oral part, dorsomedial division. Representative images (**a**–**h**) show overlap with *Slc32a1* in cerebellum (**a**–**b**, **a**’–**b**’) or *Slc17a6* (**c**–**d**, **c**’–**d**’) and *Slc32a1* in brainstem (**e**–**f**, **e**’–**f**’) or *Slc17a6* (**g**–**h**, **g**’–**h**’), enlargements marked with dashed boxes. White arrows mark examples of double-positive cells, and magenta arrows mark examples of *Slc6a5*-only cells (≥ 5 dots within the same cell). Scale bars: 100 μm, enlargements 50 μm. Separate channels can be found in Additional file 1: Fig. S6

In the more caudal brain regions, RNAscope results were consistent with previous findings [32, 66, 67], with high expression observed in the cerebellum and brainstem (Fig. 9a–h). In both female and male mice, the majority of *Slc6a5* labelled cells in the cerebellum co-expressed *Slc32a1* (female – 89 ± 3%, range of analysed sects. 78–97%, *n* = 2 mice, 2 sections per mouse; male – 94 ± 1%, range of analysed sects. 87–99%, *n* = 2 mice, 2–3 sections per mouse) (Fig. 9a b), whereas almost no co-expression between *Slc6a5* and the excitatory marker *Slc17a6* was detected (female – 0.2 ± 0%, range of analysed sects. 0–0.3%, *n* = 2 mice, 2 section per mouse; male – 0 ± 0%, (range of analysed Sect. 0%, *n* = 2 mice, 2–3 sections per mouse) (Fig. 9c, d).

In the brainstem, RNAscope results aligned with scRNA-seq and qRT-PCR findings (Fig. 9e–h). Similar to the cerebellum, most *Slc6a5* labelled cells in the brainstem were co-labelled with the inhibitory marker (female – 96 ± 2%, range of analysed sects.  88–99%, *n* = 2 mice, 1–3 sections per mouse; male – 96 ± 0.1%, range of analysed sects.  93–99%, *n* = 2 mice, 2–3 sections per mouse) (Fig. 9e, f). However, unlike the cerebellum, where no *Slc17a6* co-expression was observed, approximately 20–50 *Slc6a5*-labelled/*Slc17a6*-labelled cells per section were identified in both female and male mice (female – 2 ± 0%, range of analysed sects.  1– 3%, *n* = 2 mice, 2 sections per mouse); male: 2 ± 0.2%, range of analysed sects.  1–4%, *n* = 2 mice, 2–3 sections per mouse) (Fig. 9g, h).

**Fig. 10:**
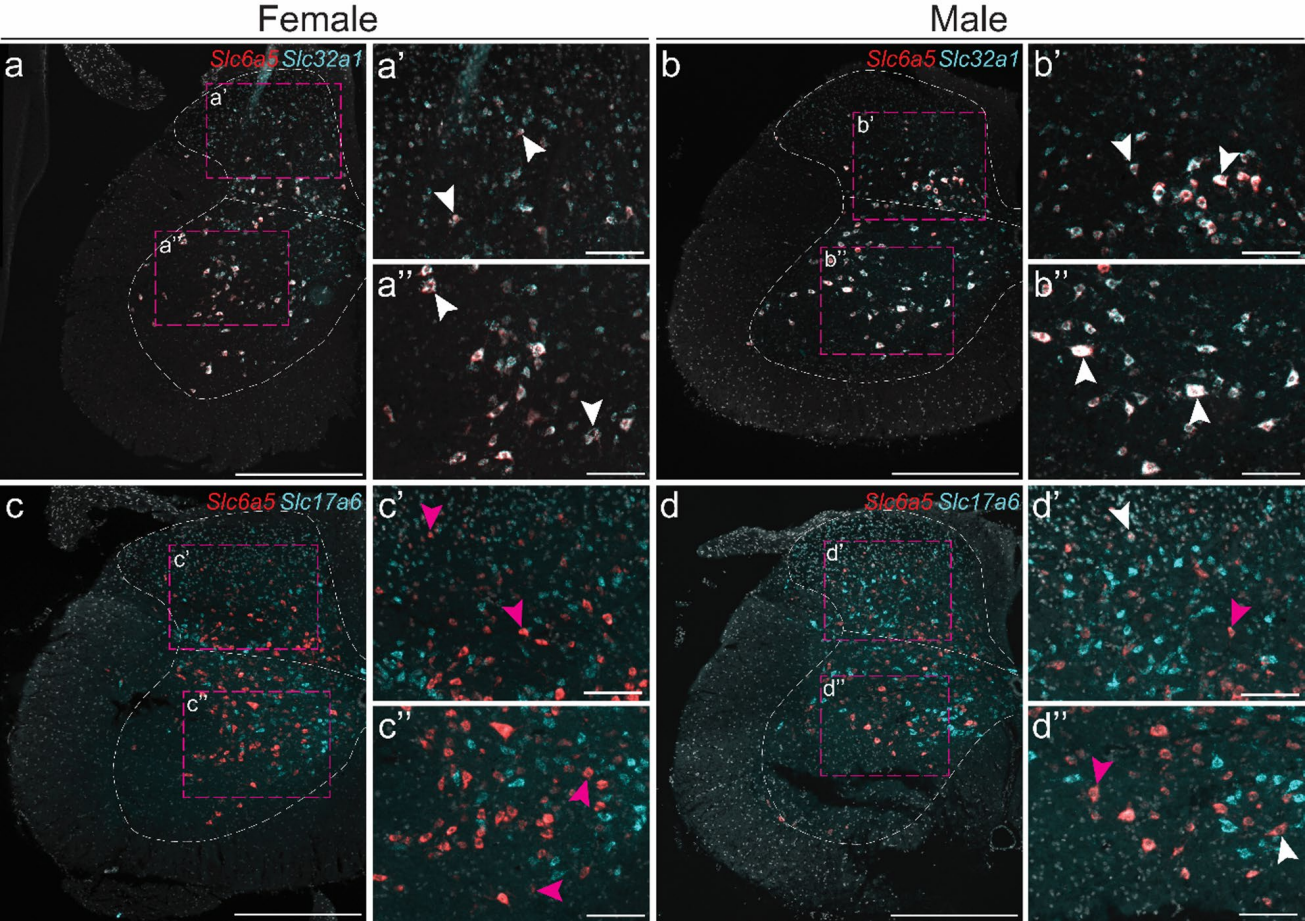
*Histological analysis of the spatial expression of Slc6a5 in lumbar spinal cord.* RNAscope was performed on (**a**, **c**) L4–L5 sections from female mice and (**b**, **d**) L4–L5 sections from male mice. (**a**–**d**) Representative images of the lumbar spinal cord show *Slc6a5* (red), the inhibitory marker *Slc32a1* (light blue), the excitatory marker *Slc17a6* (light blue), and nuclei staining with DAPI (light grey). Dashed boxes indicate areas shown in enlargements (**a**’–**d**’’). White arrows mark examples of double-positive cells, while magenta arrows mark *Slc6a5*-only cells. Scale bars: 100 μm, enlargements 50 μm

GLYT2-Cre cells in the spinal cord have been demonstrated to play a prominent role in the regulation of sensory information [68]. Spatial analysis using RNAscope revealed that *Slc6a5*-labelled cells could be found in both the dorsal and ventral part in all divisions of the spinal cord in both female and male mice (Fig. 10a–d, Additional file 1: Fig. S7–S11). Moreover, *Slc6a5* expression was detected in a small subset of DRG cells (Fig. 7b), which contrasted with the scRNA-seq data (Figs. 1 and 2). This discrepancy may be attributable to technical limitations, as no *Slc6a5*-positive cells were captured during sample preparation or the expression of *Slc6a5* was below the detection threshold in the Zeisel et al. (2018) dataset [42].

Previous studies have suggested that *Slc6a5* functions as an inhibitory marker for glycinergic neurons [69, 70], and this interpretation is largely supported by our spinal cord data. The majority of *Slc6a5*-positive cells were found to co-express *Slc32a1 *in both the dorsal (female – 68 ± 1%, range of analysed sects. 32–87%, *n* = 2 mice, 11 sections per mouse; male – 63 ± 0.3%, range of analysed sects. 24–88%, *n* = 2 mice, 11 sections per mouse) and ventral part (female – 71 ± 2%, range of analysed sects. 35–98%, *n* = 2 mice, 11 sections per mouse; male – 66 ± 0.1%, range of analysed sects. 29–86%, *n* = 2 mice, 11 sections per mouse) of the spinal cord, a pattern that was consistent across all spinal cord divisions (Fig. 10a, b, Additional file 1: Fig. S7–S9). Nevertheless, a small subset of *Slc6a5*-positive cells co-expressed *Slc17a6*, the gene encoding vesicular glutamate transporter 2 (VGLUT2), in both the dorsal (female – 5 ± 0.1%, range of analysed sects. 2–19%, *n* = 2 mice, 8 sections per mouse; male – 5 ± 0.5%, range of analysed sects. 0–16%, *n* = 2 mice, 8 sections per mouse) and ventral (female – 6 ± 1%, range of analysed sects. 1–7%, *n* = 2 mice, 8 sections per mouse; males – 4 ± 0.1%, range of analysed sects. 0–12%, *n* = 2 mice, 8 sections per mouse) part of the spinal cord from female and male mice (Fig. 10c, d, Additional file 1: Fig. S7–S9), indicating that *Slc6a5* is not exclusively associated with inhibitory populations. This observation aligns with findings from a larger RNA-sequencing study conducted by Häring and colleagues [71].

## Discussion

Here, we present a comprehensive analysis of *Slc6a9* (GLYT1) and *Slc6a5* (GLYT2) mRNA expression using three complementary mRNA-based approaches in female and male mice (Table 2). During the early 2010s, members of the SLC6 transporter family were systematically characterized and demonstrated to be conserved across vertebrates, where they are involved in essential physiological processes [72], including amino acid transport, osmotic homeostasis, and modulation of neurotransmitter signalling in both the peripheral and central nervous systems [73]. Beyond these roles, SLC6 transporters have been implicated in spermatogenesis [74], and in the intestine and kidneys they are considered critical for amino acid absorption and osmolyte reabsorption [17], underscoring their widespread expression. More recently, interest in glycine transporters as therapeutic targets has increased [5, 75–79]. Accordingly, gene expression data highlighting sex-dependent differences in a translational model organism may provide important insights for the development of safer and more precise treatment strategies.

**Table 2:** Summary of *Slc6a9* and *Slc6a5* expression in the central nervous system. Expression was considered present if values met or exceeded the following cut-offs: scRNA-seq log1p ≥ 0.1; qRT-PCR significantly expressed compared with negative control (excluding biological outliers); RNAscope ≥ 5 dots within the same cell. Symbols used: + Detected; - not detected; NS not significant against background levels, although one or more biological replicates showed expression; dark grey area not analysed

Tissue	*Slc6a9*					*Slc6a5*				
	scRNA-seq	qRT-PCR		RNAscope		scRNA-seq	qRT-PCR		RNAscope	
		♀	♂	♀	♂		♀	♂	♀	♂
Cortex	+	NS	NS	+	+	+	-	-	-	-
Amygdala	+	NS	NS	+	+	-	-	-	-	-
Striatum	+	+	NS	-	-	-	-	+	-	-
Hypothalamus	+	+	NS	+	+	-	-	-	-	-
Thalamus	+	+	+	+	+	+	-	-	+	+
Hippocampus	+	NS	NS	-	-	-	-	-	-	-
Midbrain	+					+			+	+
Cerebellum	+	+	+	+	+	+	-	+	+	+
Brainstem	+	+	+	+	+	+	+	+	+	+
Spinal cord	+	+	+	+	+	+	+	+	+	+
DRG	+			+	+	-			+	+

### The SLC6A9 and SLC6A5 expression patterns are similar across mammals

GLYT1 (*SLC6A9*) exhibits a broad expression profile across the human body. According to the HPA (https://www.proteinatlas.org/ENSG00000196517-SLC6A9/tissue) [63], both mRNA and protein expression have been detected in a wide range of tissues, including the brain, eye, endocrine organs, respiratory system, gastrointestinal tract, liver, pancreas, kidney, reproductive tissues (male and female), muscle, connective and soft tissues, skin, bone marrow, and lymphoid tissues. Furthermore, *SLC6A9* mRNA has been identified in human enterocytes of the intestine [80], and in the prefrontal cortex [81]. This widespread distribution indicates that GLYT1 (https://www.proteinatlas.org/ENSG00000196517-SLC6A9/tissue) [63] potentially has physiological roles outside of the central nervous system. One such potential role that has been established is the link between glycine transporter 1 and haemoglobinization and iron homeostasis [82–84]. Another known physiological function where GLYT1 has a role is in the absorption and reabsorption of amino acids in the intestine and kidneys, maintaining systemic glycine homeostasis [85, 86]. Our bulk-tissue analysis showed sex-dependent differences in the mRNA expression of *Slc6a9* in the intestine and kidneys, suggesting that there might be sex‑specific regulatory mechanisms governing glycine transport and homeostasis in peripheral tissues. Single-cell RNA sequencing demonstrated a broad range of cell types expressing *SLC6A9*, with particularly enhanced expression in horizontal cells, erythroid cells, bipolar cells, distal enterocytes, and suprabasal keratinocytes [87, 88]. Consistent with earlier studies, recent single-nucleus RNA sequencing has revealed enriched *SLC6A9* expression in glial populations, particularly astrocytes, ependymal cells, and oligodendrocytes [87, 88], coherent with our findings in the re-analysis of the Zeisel et al. (2018) dataset. In rodents, *Slc6a9* expression has been detected in the developing mouse spinal cord and in the adult rat liver, lung, spinal cord, cerebellum, hippocampus, and optic nerve [29]. These findings are consistent with our results, in which all three mRNA-detection methods consistently revealed widespread *Slc6a9* expression in mice.

According to the HPA, RNA sequencing has revealed that *SLC6A5* expression in humans is relatively limited, with mRNA detection in the caudal brain, endocrine tissues, respiratory system, and testis (https://www.proteinatlas.org/ENSG00000165970-SLC6A5/tissue) [63]. Mutations in the human *SLC6A5* gene have been implicated in hyperekplexia [89]. Within the central nervous system, *SLC6A5* has primarily been localized to inhibitory neurons [69, 70, 78]. Interestingly, GLYT2 has been identified in excitatory neurons of the pre-Bötzinger complex in rats [90], in the pontine reticular formation and PAG in mice [91, 92], and in the cochlear nuclei of humans and chimpanzees [93]. In addition, single-nucleus RNA analysis of the human brain demonstrated expression in inhibitory neurons of the cerebellum, the hippocampal CA4 region, and medial ganglionic eminence interneurons [87, 88]. These findings are consistent with our data, which indicates a predominantly caudal expression pattern in the mouse brain. However, *Slc6a5* expression was not detected in the hippocampal CA regions. It should be noted that mice lack the CA4 region; nevertheless, we observed expression in close proximity to the dentate gyrus.

Beyond the nervous system, expression has been reported in human islet β-cells [94, 95]. Single-cell RNA sequencing has revealed that *SLC6A5* is expressed in spermatids, oocytes, pituitary gland cells, and adrenal cortex cells (https://www.proteinatlas.org/ENSG00000165970-SLC6A5/single+cell). In visceral organs, *Slc6a5* expression was detected by qRT-PCR in some biological replicates of intestine and testis, which is in line with previous reports [88].

### Slc6a9 can be found in both neurons and glial cells

*Slc6a9* has primarily been reported as expressed in glial cells located near glycinergic neurons [55, 62]. However, our combined in situ hybridization and immunohistochemistry showed that *Slc6a9* is expressed in both NEUN-positive (neurons) and GFAP-positive (astrocytes) cells. This observation is consistent with previous RNA sequencing analyses [87, 88], and our re-analysis of the Zeisel et al. (2018) dataset [42] where *Slc6a9* expression was found in neurons (Figs. 1 and 2). Moreover, *Slc6a9* expression was identified in cells that did not express NEUN or GFAP, indicating additional *Slc6a9*-positive cellular subpopulations. This finding is further supported by earlier RNA sequencing data [42, 87, 88]. Importantly, NEUN does not label all neurons [96–101]. While it is a robust marker for cortical neurons, hippocampal CA1–CA3 neurons, cerebellar granule cells, and neurons of the brainstem and spinal cord, NEUN fails to label certain inhibitory or specialized neuronal populations, such as cerebellar Purkinje cells, olfactory bulb mitral cells, and subsets of interneurons in the cortex and hippocampus [96–101]. Members of the SLC6 family are secondary active co-transporters that utilize the sodium gradient to transport their substrates, and in some cases chloride ions [17, 19, 102]. Both GLYT1 and GLYT2 transport sodium ions, chloride ions, and glycine, but their biochemistry differs. GLYT1 couples glycine transport with two sodium ions and one chloride ion, whereas GLYT2 couples glycine transport with three sodium ions and one chloride ion [103]. Variances in transport stoichiometries may reflect their differences in expression patterns, with GLYT1 is primarily glial, whereas GLYT2 is neuronal [103].

### Sex-dependent differences in expression of Slc6a5

Recently, Jiang et al. (2025) investigated the sex-specific distribution of GLYT2 neurons in the central nervous system of mice using the GlyT2-iCre mouse line in combination with the tdTomato reporter line [67]. Non-oestrus females were reported to exhibit higher GLYT2-tdTomato expression across 12 brain regions within the thalamus, midbrain, and hindbrain compared with males, whereas males showed higher expression in the ventral posteromedial nucleus and lateral cerebellar regions [67]. Our results for *Slc6a5* indicated generally higher expression in males based on the bulk-tissue analysis using qRT-PCR (the cortex, striatum, hippocampus, and spinal cord), which is inconsistent with their tdTomato findings. However, it should be noted that tdTomato expression may not fully represent the adult GLYT2 population, which has been the focus of our study, as distinguishing between developmentally labelled and mature GLYT2 neurons remain challenging, a limitation acknowledged by Jiang et al. (2025) [67]. In addition, the authors performed RNAscope against *Slc6a5* in male mice, where they observed a sparser expression pattern, consistent with our findings. RNAscope labelling was reported in the thalamus, midbrain, and hindbrain, regions in which expression was replicated in our study.

### Slc6a9 and Slc6a5 co-localize with the glutamate transporter Slc17a6 in several brain regions

Most neurons expressing *Slc6a9* or *Slc6a5* were found to co-express *Slc32a1*, indicating that these cells are inhibitory. Glycine is released to hyperpolarize the postsynaptic neuron via interactions with pentameric ligand-gated ion channels [1–5] and the metabotropic glycine receptor, previously referred to as *Gpr158* [104]. However, in certain regions, including the hypothalamus and caudal structures such as the midbrain, medulla, and spinal cord, *Slc6a9* was expressed in comparable proportions across both excitatory and inhibitory neurons. In contrast, within the thalamus, pons, and DRG, *Slc6a9* predominantly co-localized with the excitatory glutamatergic transporter *Slc17a6*. Comparable patterns were observed in the thalamic neurons during the *Slc6a5* analysis. This demonstrates that subpopulations of *Slc6a9*- or *Slc6a5*-expressing neurons are excitatory and can utilize glutamate to influence the postsynaptic membrane. In this setting, the classical inhibitory transmitter glycine assumes an excitatory role by binding to its co‑agonist site on NMDA receptors, thereby enhancing receptor activation in the presence of glutamate or *N*-methyl-D-aspartate (NMDA) ([105], reviewed by [106]). This potentiation occurs through an increased frequency of NMDA channel opening [105]. Consequently, the presence of *Slc6a9* in excitatory synapses may serve to regulate glutamatergic tone [107].

### Methodological consideration

Minor discrepancies between the three analyses, as noted previously [43, 44], are likely attributable to differences in sample preparation and assay sensitivity. In the scRNA-seq dataset generated by Zeisel et al. (2018) [42], which represents one of the most comprehensive single-cells transcriptomic maps of the mouse nervous system, samples from both females and males were pooled during tissue dissociation and library preparation. This means that the resulting dataset represents a composite transcriptomic profile that precludes the disaggregation of sex-specific expression differences. A key distinction exists between the genetic foundations of the Zeisel et al. (2018) dataset [42], where a heterogenous mix of outbred (CD-1, Swiss) and mixed background (CD-1, C57BL/6J) transgenic mice was employed to capture a broad landscape of cellular diversity, the rest of our analysis was conducted exclusively on the inbred C57BL/6J strain. By utilizing a strictly inbred model, the background noise and genetic variability inherent in outbred populations were minimized, providing a highly controlled baseline for *Slc6a9* and *Slc6a5* expression [108–110]. However, it is important to note that the high-resolution cell-type mapping in the Zeisel et al. (2018) dataset might reflect a wider range of physiological states that are more homogenized in a pure C57BL/6J cohort. In addition to strain differences, the temporal windows of the Zeisel et al. (2018) dataset [42] and our analysis offer distinct physiological perspectives. The scRNA-seq dataset incorporates a developmental range, including juvenile postnatal days (P12–P30) and early adult stages (6–8 weeks), which may capture transient high-expression levels of *Slc6a9* and *Slc6a5* associated with late-stage maturation of the nervous and peripheral systems [111, 112]. In contrast, our bulk-tissue and spatial analysis utilized mice aged 10–14 weeks, representing a stable phase of biological maturity [113–115]. This allows our findings to reflect the glycine transporters’ expression in a steady-state adult physiology, though it may exclude the heightened plastic or developmental expression patterns present in the earlier time-points analysed by Zeisel et al. (2018). Furthermore, because the dataset was derived from captured single cells [42], certain cell populations may have been lost during sample preparation, which may be one of the reasons why the re-analysis of the scRNA-seq dataset and our RNAscope data are inconsistent, particularly in the DRG analysis of *Slc6a5* expression. However, the discrepancy in age could also be a contributing factor. By contrast, qRT-PCR analysis was performed on bulk-prepared samples, in which whole tissue or larger regions were collected. This approach has both advantages and limitations. On the one hand, it ensures that a broader area is sampled, reducing the likelihood of missing specific expressing cells. On the other hand, regional variability in expression can be masked, as areas with lower expression may dilute signals from regions with higher expression. As a result, qRT-PCR may fail to capture subtle, region-specific differences.

Furthermore, scRNA-seq, qRT-PCR, and RNAscope differ in sensitivity and resolution. scRNA-seq provides broad transcriptome coverage but can miss low-abundance transcripts due to dropout events. qRT-PCR is highly sensitive and quantitative for specific genes but lacks spatial resolution. RNAscope offers single-cell spatial resolution with high sensitivity but is limited to targeted transcripts and sections. These methodological differences influence whether subtle, region-specific, or sex-dependent mRNA expression patterns are detected.

## Conclusion

These findings indicate that both *Slc6a9* and *Slc6a5* are expressed across multiple brain regions, with *Slc6a9* exhibiting a broader distribution in neurons and glial cells. Furthermore, both transporters were predominantly found to co-express the inhibitory vesicular amino acid transporter, especially *Slc6a5*; however, in certain regions co-expression with the excitatory vesicular glutamate transporter 2 was found. In regions where sex-dependent differences were observed, using qRT-PCR, males generally displayed higher expression levels than females, suggesting a potential role for sex-specific regulation of glycine transporters in cellular function and signalling.

## Supplementary Information

Below is the link to the electronic supplementary material.

Supplementary Material 1

## Data Availability

The scRNA-seq data was acquired from Zeisel et al. (2018) scRNA-seq dataset [42], where the raw sequence data is deposited in the sequence read archive under accession SRP135960, available at https://www.ncbi.nlm.nih.gov/sra/SRP135960. The dataset ‘l5_all.loom’ was acquired from (http://linnarssonlab.org/). The dataset was analyzed using SCANPY 1.9.1 [45] in Python 3.8.8 in similarity as described before [43, 44, 46] and the full code can be found at https://github.com/HannahMWeman/glra3-expression-analysis-in-the-nervous-system with minor changes (*Glra3 *was changed to *Slc6a9* or *Slc6a5*) and https://github.com/MikaelaCeder/analysis-glycine-transporter-expression.ipynb. All data generated or analyzed during this study are included in this published article and its additional files.

## Declarations

### Generative AI and AI-assisted technologies in the writing process

During the preparation of this work the authors used Microsoft 365 Copilot in order to improve the readability and language of the manuscript. After using this tool, the authors reviewed and edited the content as needed and take full responsibility for the content of the published article.

## Abbreviations

*Actb*: Actin-related protein 1B
BLAST: Basic Local Alignment Search Tool
CA: Cornu Ammonis
Ct: Cycle threshold
*Cyclo*: Peptidylprolyl isomerase A
DAPI: 4′,6-diamidino-2-phenylindole
DRG: Dorsal root ganglia
FISH: Fluorescent in-situ hybridization
*Gapdh*: Glyceraldehyde-3-phosphate dehydrogenase
GFAP: Glial Fibrillary Acidic Protein
GLYT: Glycine transporter
GLYT1: Glycine transporter 1
GLYT2: Glycine transporter 2
*Gpr158*: G-protein coupled receptor 158
HPA: Human protein atlas
KW: Kruskal-Wallis
mRNA: Messenger ribonucleic acid
NEUN: Neuronal Nuclei
NMDA: N-methyl-D-aspartate
PAG: Periaqueductal Grey
PBS: Phosphate-buffered saline
PBST: Phosphate-buffered saline with Trition-X
qRT-PCR: Quantitative reverse transcription polymerase chain reaction
*Rpl19*: Ribosomal protein L19
ScRNA-seq: Single-cell ribonucleic acid sequencing
SLC6: Solute carrier family 6
*Slc6a5*: Solute carrier family 6, member a5
*Slc6a9*: Solute carrier family 6, member a9
*Slc17a6*: Solute carrier family 17, member a6
*Slc32a1*: Solute carrier family 32, member a1
VGLUT2: Vesicular glutamate transporter 2
VIAAT: Vesicular inhibitory amino acid transporter
