# Supplementary material for: Slc6a9 is distributed in glial cells and neurons across several nervous system regions, whereas Slc6a5 is more restricted to neurons in the caudal brain
Source: BMC Neurosci. 2026 Feb 17;27:12. doi: 10.1186/s12868-026-00999-3 (PMC12931055; doi:10.1186/s12868-026-00999-3)
Supplement: Supplementary file 1 — Supplementary Material 1 [file 12868_2026_999_MOESM1_ESM.pdf]

Additional file

# ***Slc6a9* is distributed in glial cells and neurons across several nervous system regions, whereas *Slc6a5* is more restricted to neurons in the caudal brain**

Mikaela M. Ceder[1] and Malin C. Lagerström[1*]

[1]Department of Immunology, Genetics and Pathology, Uppsala University, Uppsala, Sweden

*Corresponding author: Malin.Lagerstrom@igp.uu.se

This additional file contains Additional figure S1 to S11, and Additional Table S1, 21 pages.

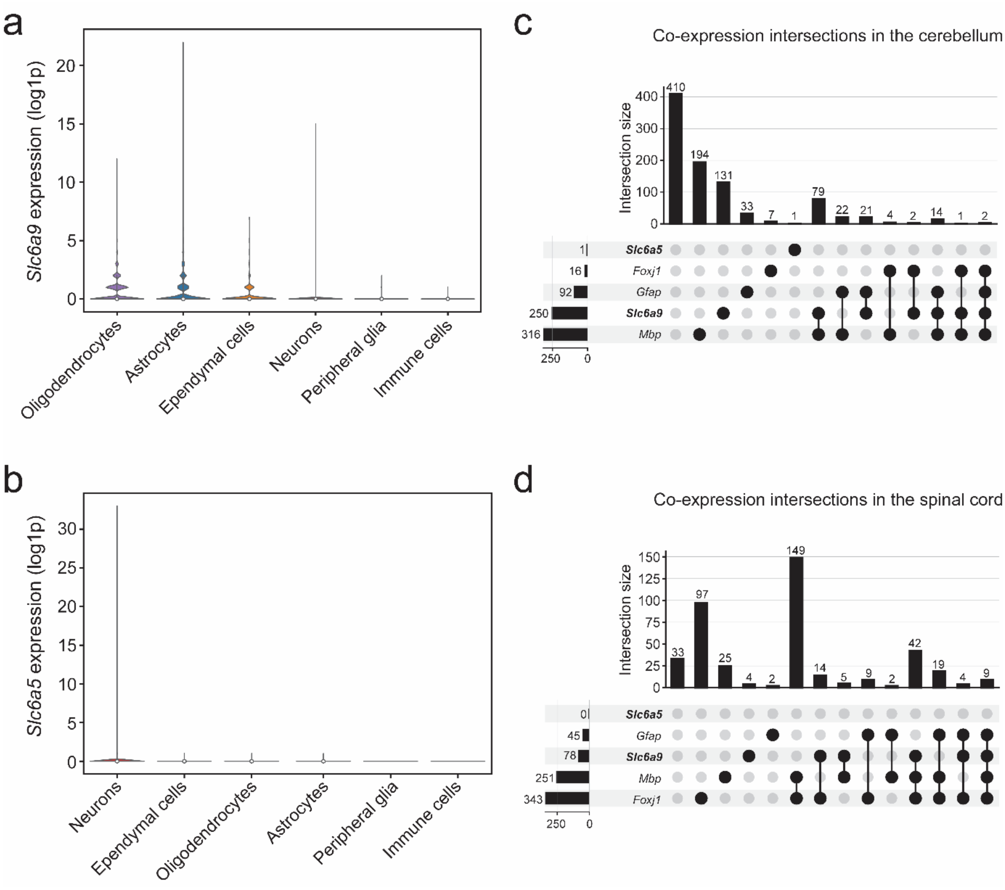


**Fig. S1, relates to fig. 2c.** *Slc6a9 and Slc6a5 expression in different annotated cell classes in the Zeisel et al. (2018) dataset.* (a–b) Violin plots showing the distribution of mRNA expression levels for (a) *Slc6a9* and (b) *Slc6a5* across major cell classes annotated by Zeisel et al. (2018) (1). Expression values are normalized and presented on a log1p scale. The order in which the cell classes appear in the plot (left to right) indicates the proportion of expression per class (from highest to lowest). (a) *Slc6a9* exhibits a multimodal expression profile, with the highest expression observed in astrocytes, followed by oligodendrocytes, ependymal cells and neurons. (b) *Slc6a5* expression is highly specific to the neuronal class, with minimal to no expression detected in non-neuronal populations. (c–d) UpSet plots showing the intersection of gene expression for *Slc6a9* and *Slc6a5* with established markers for ependymal cells (*Foxj1*), astrocytes (*Gfap*), and oligodendrocytes (*Mbp*) in the (c) cerebellum and (d) spinal cord. These two regions were chosen as they had the highest number of glial cells expressing *Slc6a9* or

*Slc6a5*. Set Size (horizontal bars) represent the total number of cells expressing each individual gene within the respective tissue. Intersection Size (vertical bars) represent the number of cells co-expressing specific combinations of genes, as indicated by the connected dots in the matrix below the bar chart.

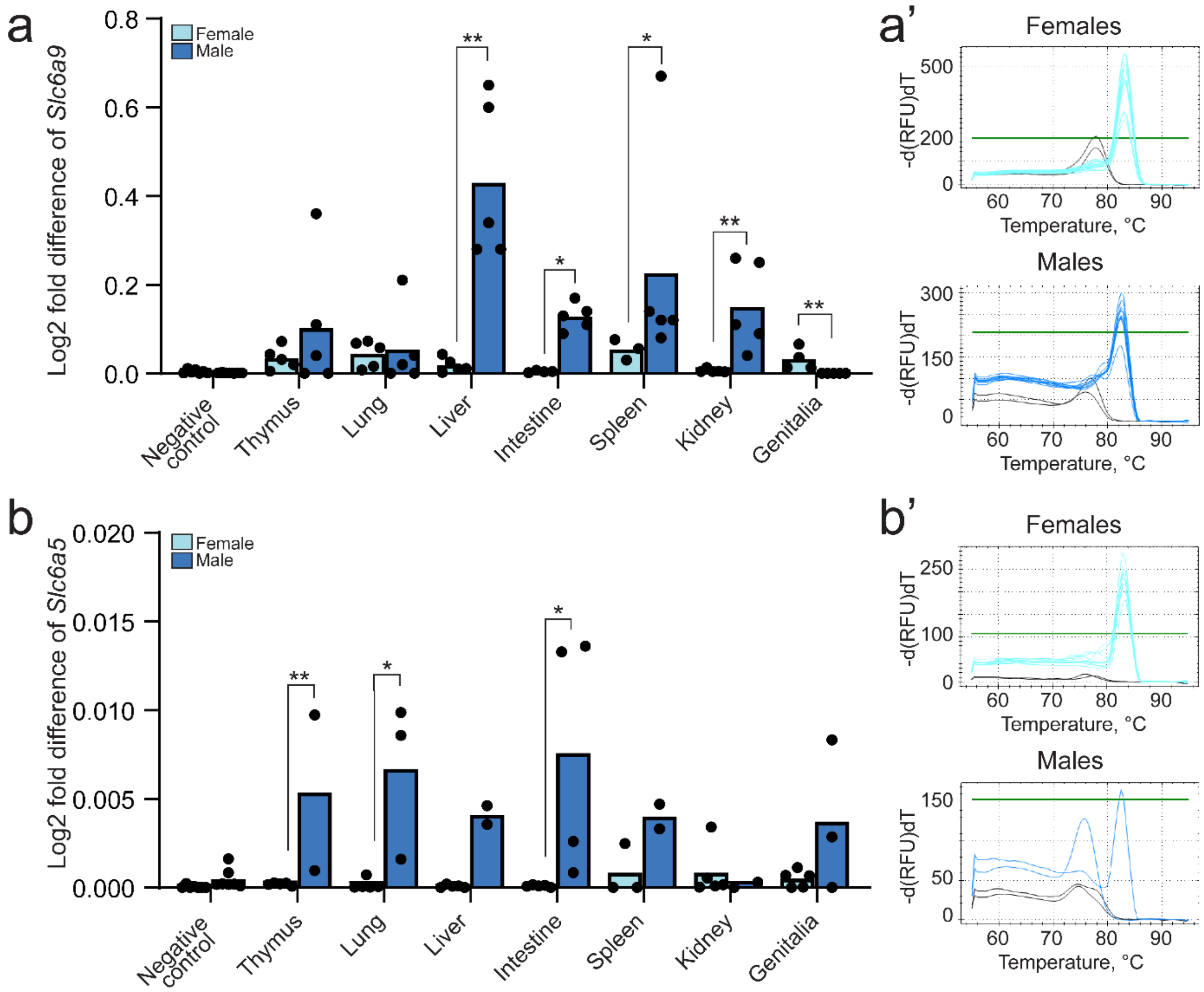


**Fig. S2, relates to fig. 3.** *Sex-dependent differences in mRNA expression of Slc6a9 and Slc6a5 in visceral organs could be detected using qRT-PCR.* Expression of *Slc6a9* and *Slc6a5* in adult female (n = 5) and adult male (n = 5) C57BL/6J mice were observed using qRT-PCR, with a cut-off of 45 cycles. Relative mRNA expression was calculated using the delta Ct method with three stable reference genes (*Cyclo*, *Rpl19*, *Gapdh*) and *Actb* was used as the calibrator. Stable reference genes were identified using the GeNorm protocol (2). Biological outliers (for *Slc6a9*: one for intestine, two for spleen and one for genitalia from female mice; for *Slc6a5*: two for spleen from female mice, three for thymus, two for lung, three for liver, one for intestine, three for spleen, three for kidney and two for genitalia from male mice) were removed using the

Grubbs outlier test with α = 0.05 prior to analysis. The $Log_2$ fold mean difference, with individual dots representing individual mice relative to *Actb* expression, is illustrated in the combined scatter-bar plot. Normality was assessed using the Shapiro-Wilk test. Difference between female and male mice for each tissue were calculated using a two-tailed Mann–Whitney U-test, where $*p < 0.05$, $**p < 0.01$. (a) *Slc6a9* expression in visceral organs of females and males, with differences in each region investigated: thymus (p=0.9365), lung (p=0.5079), liver (p=0.0079), intestine (without outlier p=0.0159; with outlier p=0.0079), spleen (without outlier p=0.0179; with outlier p=0.0079), kidney (p=0.0079), and genitalia (without outlier p=0.0079; with outlier p=0.0476). (b) *Slc6a5* expression in visceral organs of females and males, with differences in each region investigated: thymus (without outlier p=0.0952; with outlier p=0.0079), lung (without outlier p=0.0357; with outlier p=0.0079), liver (without outlier p=0.0952; with outlier p=0.1508), intestine (without outlier p=0.0159; with outlier p=0.0079), spleen (without outlier p=0.200; with outlier p=0.3095), kidney (without outlier p=0.5714; with outlier p=0.3095), and genitalia (without outlier p=0.5714; with outlier p=0.1508). Representative melt peak images for females (light blue), males (blue) and negative controls (black) are shown in panels a'–b'.

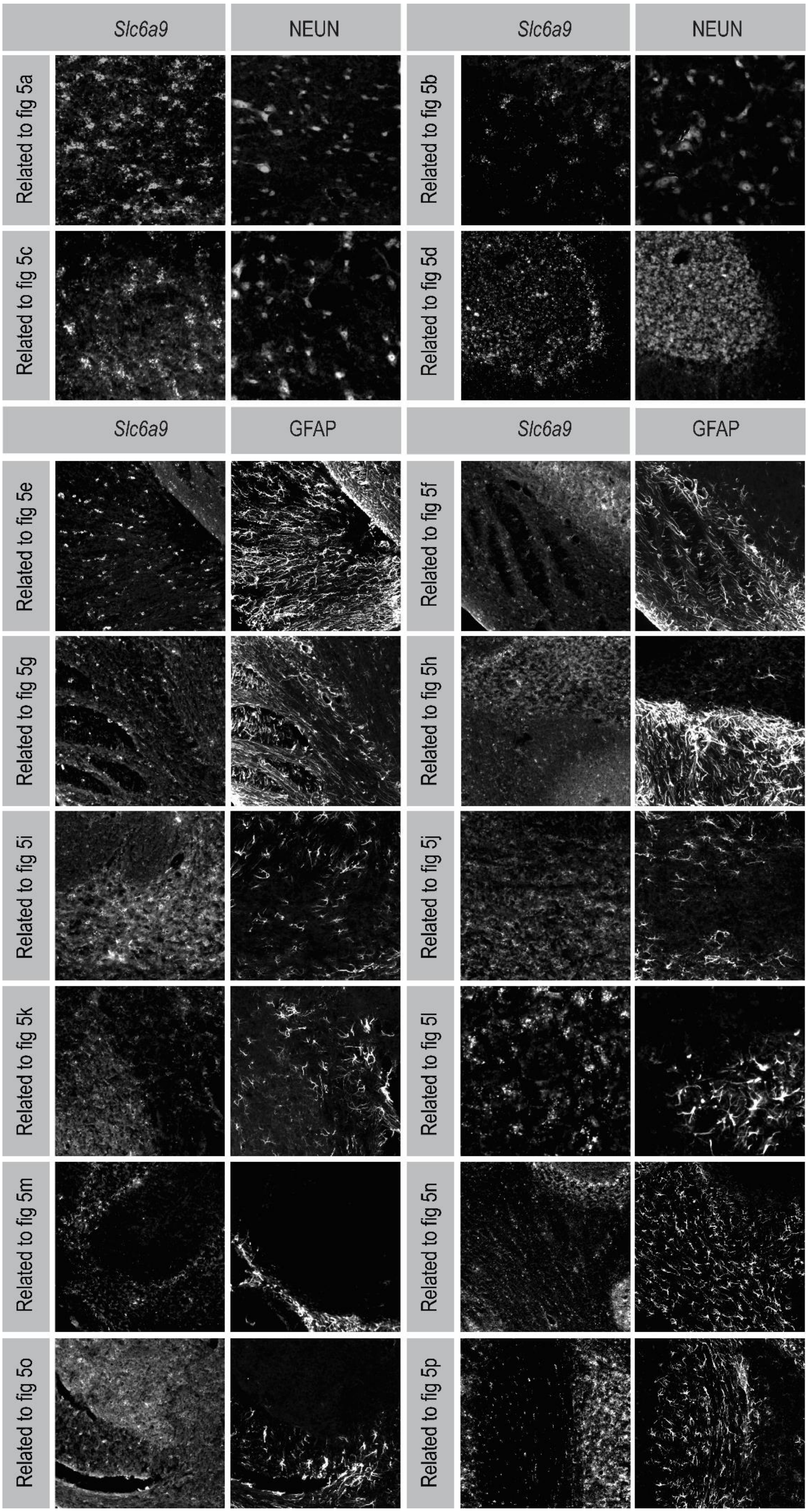

Slc6a9
NEUN
Slc6a9
NEUN
Related to fig 5a
Related to fig 5b
Related to fig 5c
Related to fig 5d
Slc6a9
GFAP
Slc6a9
GFAP
Related to fig 5e
Related to fig 5f
Related to fig 5g
Related to fig 5h
Related to fig 5i
Related to fig 5j
Related to fig 5k
Related to fig 5l
Related to fig 5m
Related to fig 5n
Related to fig 5o
Related to fig 5p

**Fig S3, relates to fig. 5.** *In situ hybridization and immunohistochemistry analysis of Slc6a9 co-expression with neuronal and astrocytic markers in the adult mouse brain*. Fluorescent RNAscope for *Slc6a9* was combined with immunohistochemistry for NEUN (neuronal marker) or GFAP (astrocytic marker). The figure displays the separate channels for *Slc6a9,* NEUN and GFAP in (grey). White arrows indicate examples of double-positive cells, while magenta arrows highlight examples of cells expressing *Slc6a9* only (≥ 5 dots within the same cell), the arrows display the same cells indicated in Fig. 5a–d. Scale bars: 100 µm.

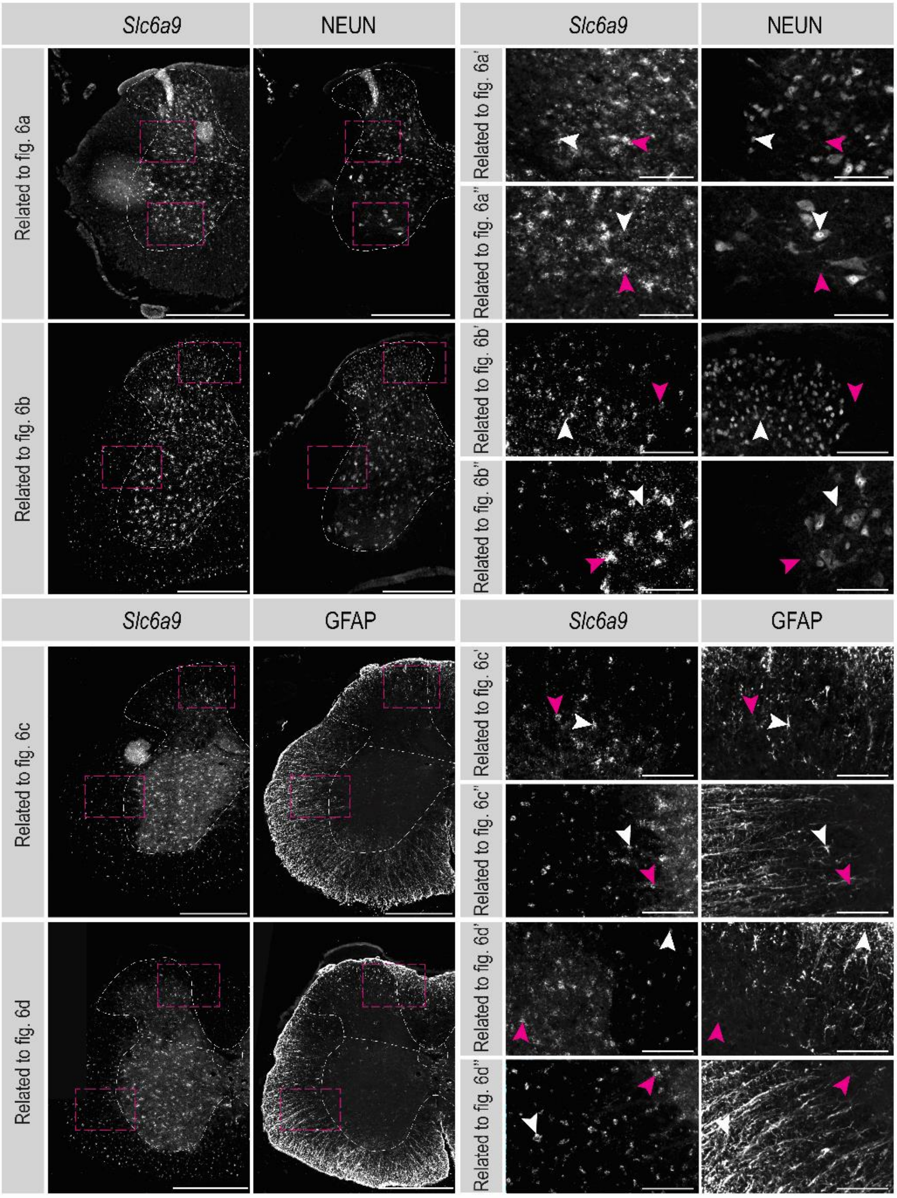


**Fig S4, relates to fig 6.** *Co-expression of Slc6a9 with neuronal and astrocytic markers in the lumbar spinal cord of adult mice.* Fluorescent RNAscope for *Slc6a9* was combined with immunohistochemistry for NEUN (neuronal marker) or GFAP (astrocytic marker). The figure displays the separate channels for *Slc6a9,* NEUN and GFAP in (grey). Dashed boxes show the position of the enlargement. White arrows indicate examples of double-positive cells, while magenta arrows highlight examples of cells expressing *Slc6a9* only ($\geq$ 5 dots within the same cell), the dashed boxes and arrows display the same areas and cells indicated in Fig. 6a'–d''. Scale bars: 500 µm; enlargements, 50 µm.

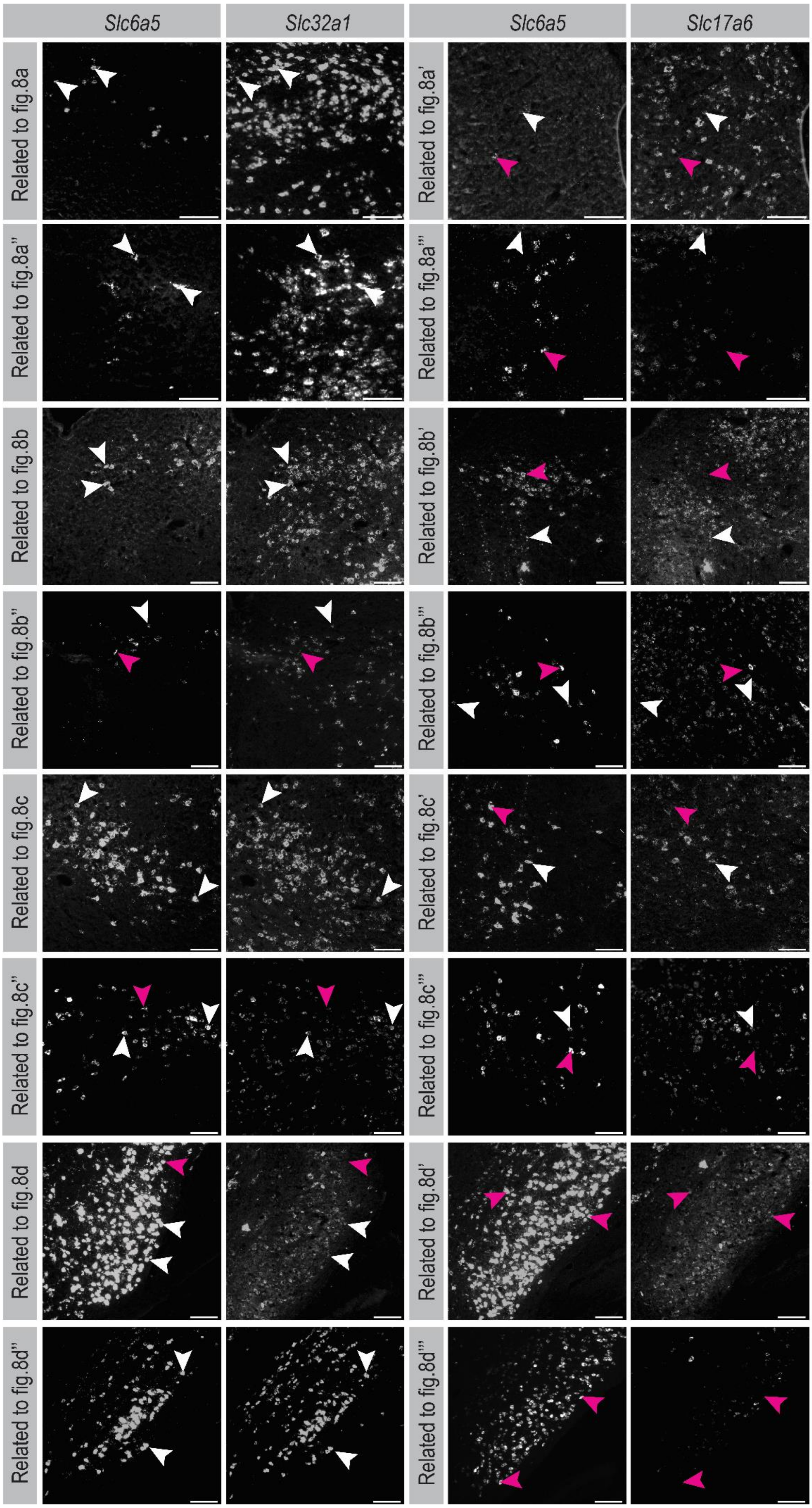

Slc6a5
Slc32a1
Slc6a5
Slc17a6
Related to fig.8a
Related to fig.8a'
Related to fig.8a''
Related to fig.8a'''
Related to fig.8b
Related to fig.8b'
Related to fig.8b''
Related to fig.8b'''
Related to fig.8c
Related to fig.8c'
Related to fig.8c''
Related to fig.8c'''
Related to fig.8d
Related to fig.8d'
Related to fig.8d''
Related to fig.8d'''

**Fig S5, relates to fig 8.** *Histological analysis of Slc6a5 expression in thalamus and midbrain, and its overlap with inhibitory and excitatory markers.* Fluorescent RNAscope for *Slc6a5* was combined with *Slc32a1* (inhibitory marker) or *Slc17a6* (excitatory marker). The figure displays the separate channels for *Slc6a9, Slc32a1 and Slc17a6* in (grey). White arrows indicate examples of double-positive cells, while magenta arrows highlight examples of cells expressing *Slc6a9* only (≥ 5 dots within the same cell), the arrows display the same cells indicated in Fig. 8a–d). Scale bars: 100 µm.

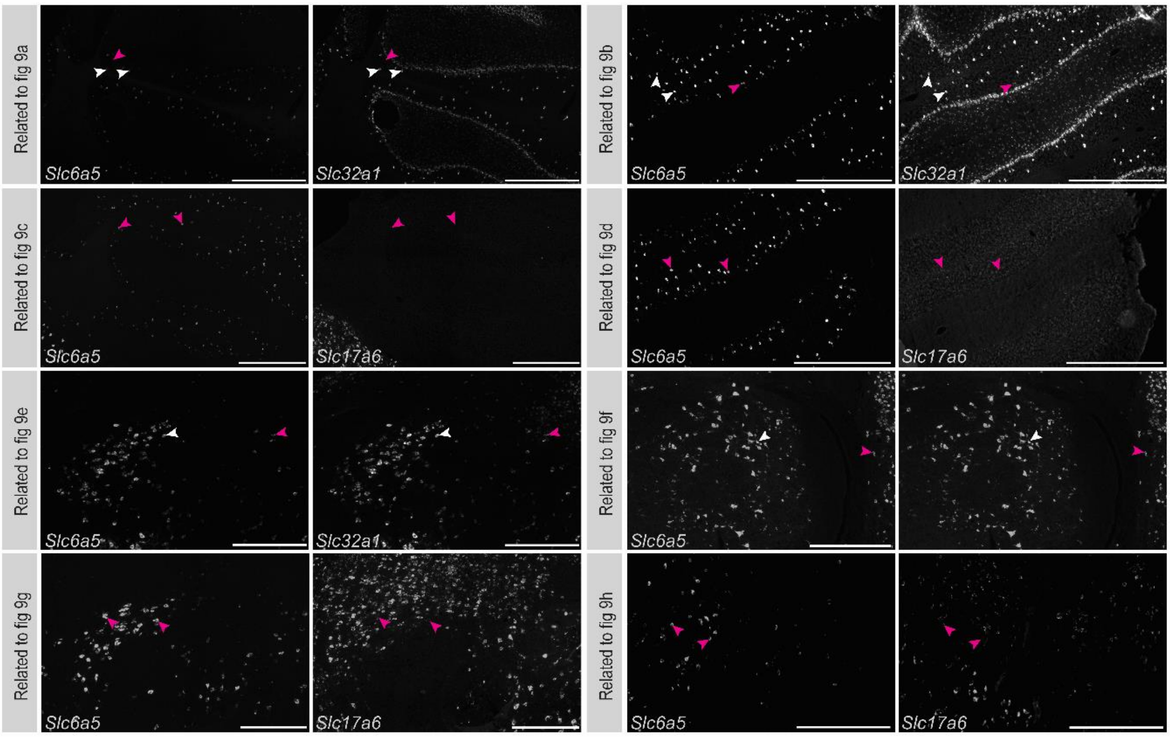


**Fig S6, relates to fig 9.** Fig 9. *Slc6a5 expression analysis reveals a predominantly inhibitory subpopulation of cells in the cerebellum and brainstem.* Fluorescent RNAscope for *Slc6a5* was combined with *Slc32a1* (inhibitory marker) or *Slc17a6* (excitatory marker). The figure displays the separate channels for *Slc6a9, Slc32a1 and Slc17a6* in (grey). White arrows indicate examples of double-positive cells, while magenta arrows highlight examples of cells expressing *Slc6a9* only (≥ 5 dots within the same cell), the arrows display the same cells indicated in Fig. 9a–d). Scale bars: 100 µm.

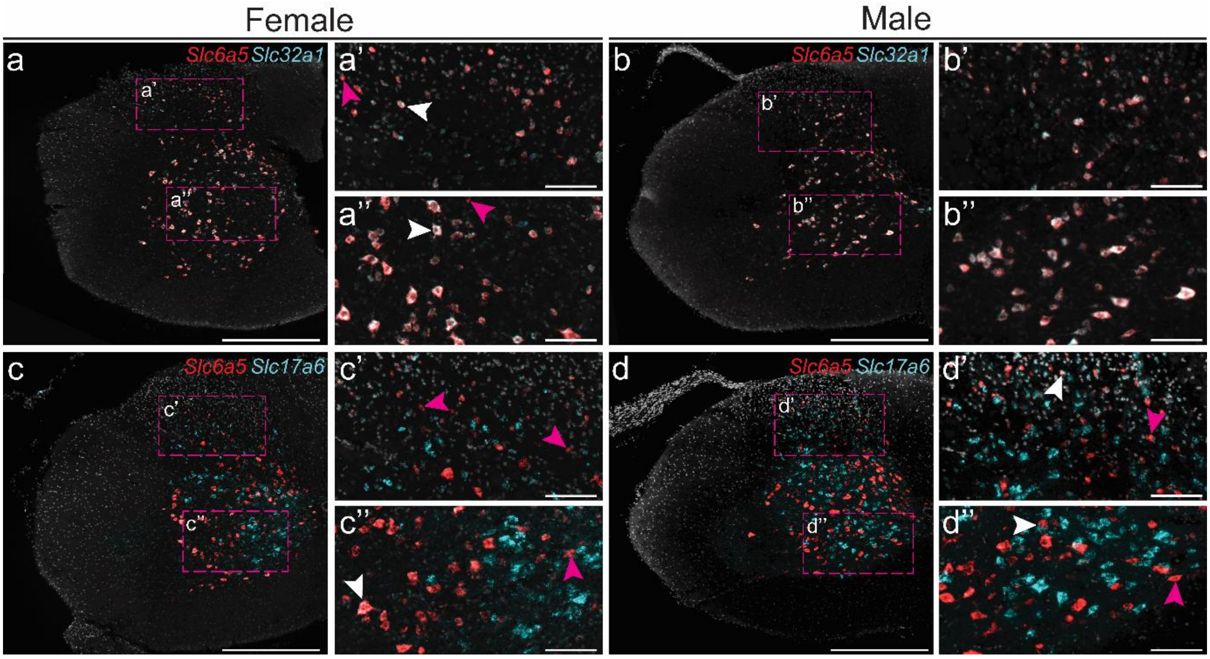


**Fig S7, relates to fig 10.** *Histological analysis of the spatial expression of Slc6a5 in the cervical spinal cord.* RNAscope was performed on (a, c) C2 sections from female mice and (b, d) C4 sections from male mice. (a–d) Representative images of the cervical spinal cord show *Slc6a5* (red), the inhibitory marker *Slc32a1* (light blue), the excitatory marker *Slc17a6* (light blue), and nuclei staining with DAPI (light grey). Dashed boxes indicate areas shown in enlargements (a'–d''). White arrows mark examples of double-positive cells, while magenta arrows indicate examples of *Slc6a5*-only cells. Scale bars: 100 µm; enlargements 50 µm.

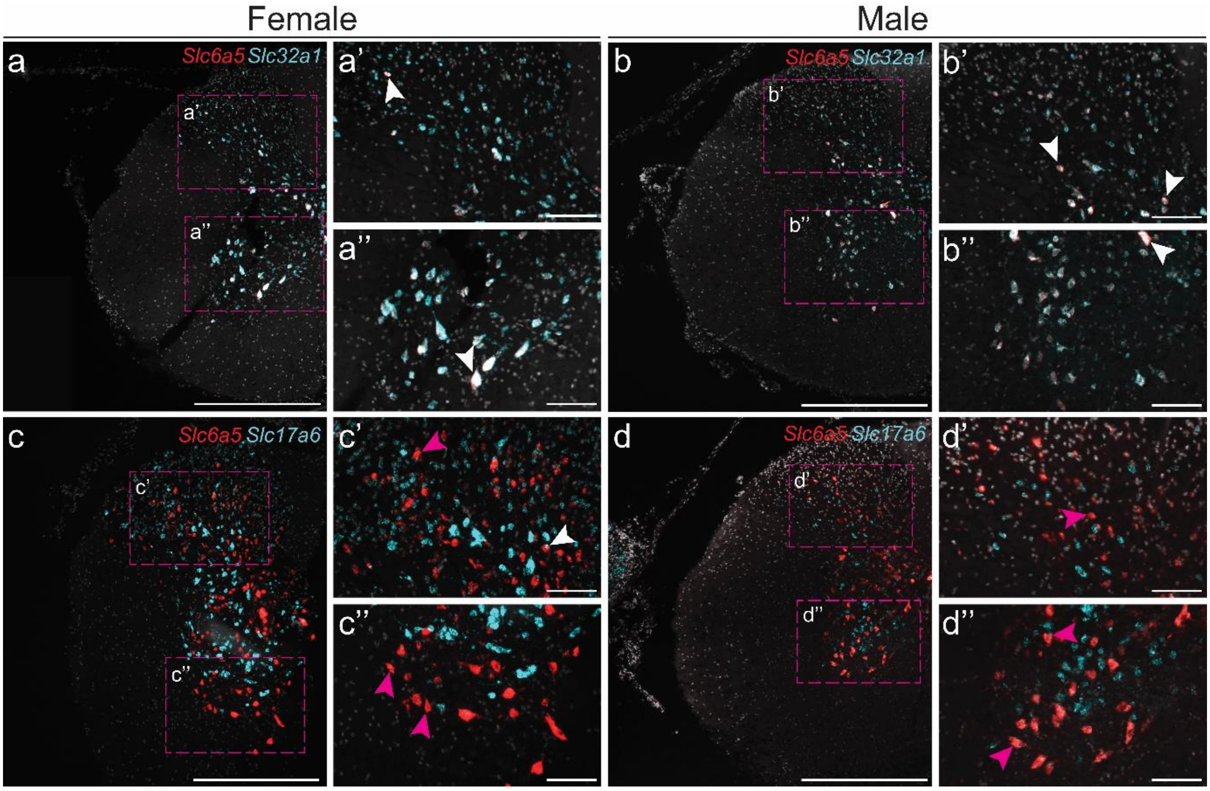


**Fig S8, relates to fig 10.** *Histological analysis of the spatial expression of Slc6a5 in the thoracic spinal cord.* RNAscope was performed on (a, c) T2 sections from female mice and (b, d) T13 sections from male mice. (a–d) Representative images of the thoracic spinal cord show *Slc6a5* (red), the inhibitory marker *Slc32a1* (light blue), the excitatory marker *Slc17a6* (light blue), and nuclei staining with DAPI (light grey). Dashed boxes indicate areas shown in enlargements (a'–d''). White arrows mark examples of double-positive cells, while magenta arrows indicate examples of *Slc6a5*-only cells. Scale bars: 100 µm; enlargements 50 µm.

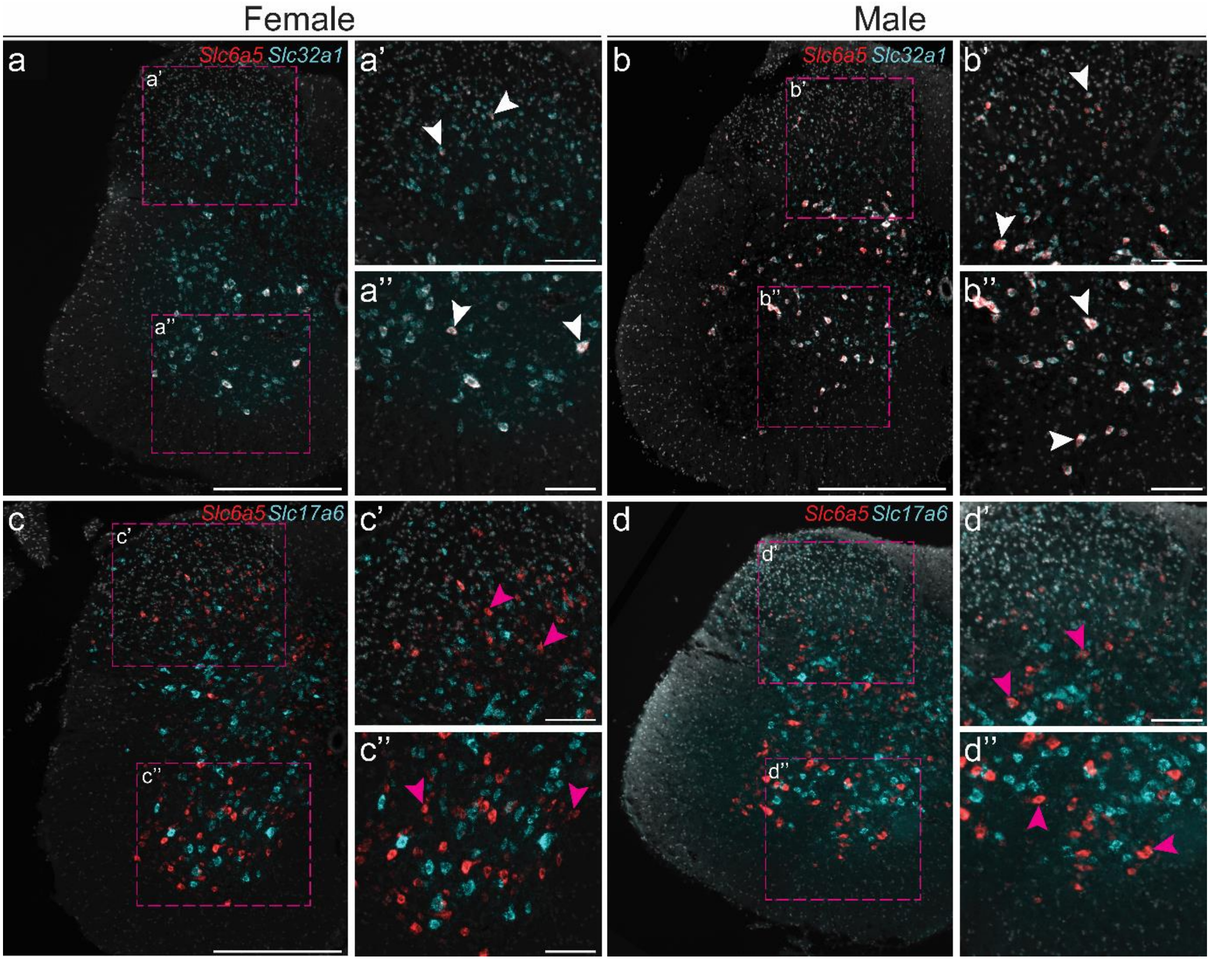


**Fig S9, relates to fig 10.** *Histological analysis of the expression of Slc6a5 in the sacral spinal cord.* RNAscope was performed on (a, c) S1 sections from female mice and (b, d) S1 sections from male mice. (a–d) Representative images of the sacral spinal cord show *Slc6a5* (red), the inhibitory marker *Slc32a1* (light blue), the excitatory marker *Slc17a6* (light blue), and nuclei staining with DAPI (light grey). Dashed boxes indicate areas shown in enlargements (a'–d''). White arrows mark examples of double-positive cells, while magenta arrows mark examples *Slc6a5*-only cells. Scale bars: 100 µm; enlargements 50 µm.

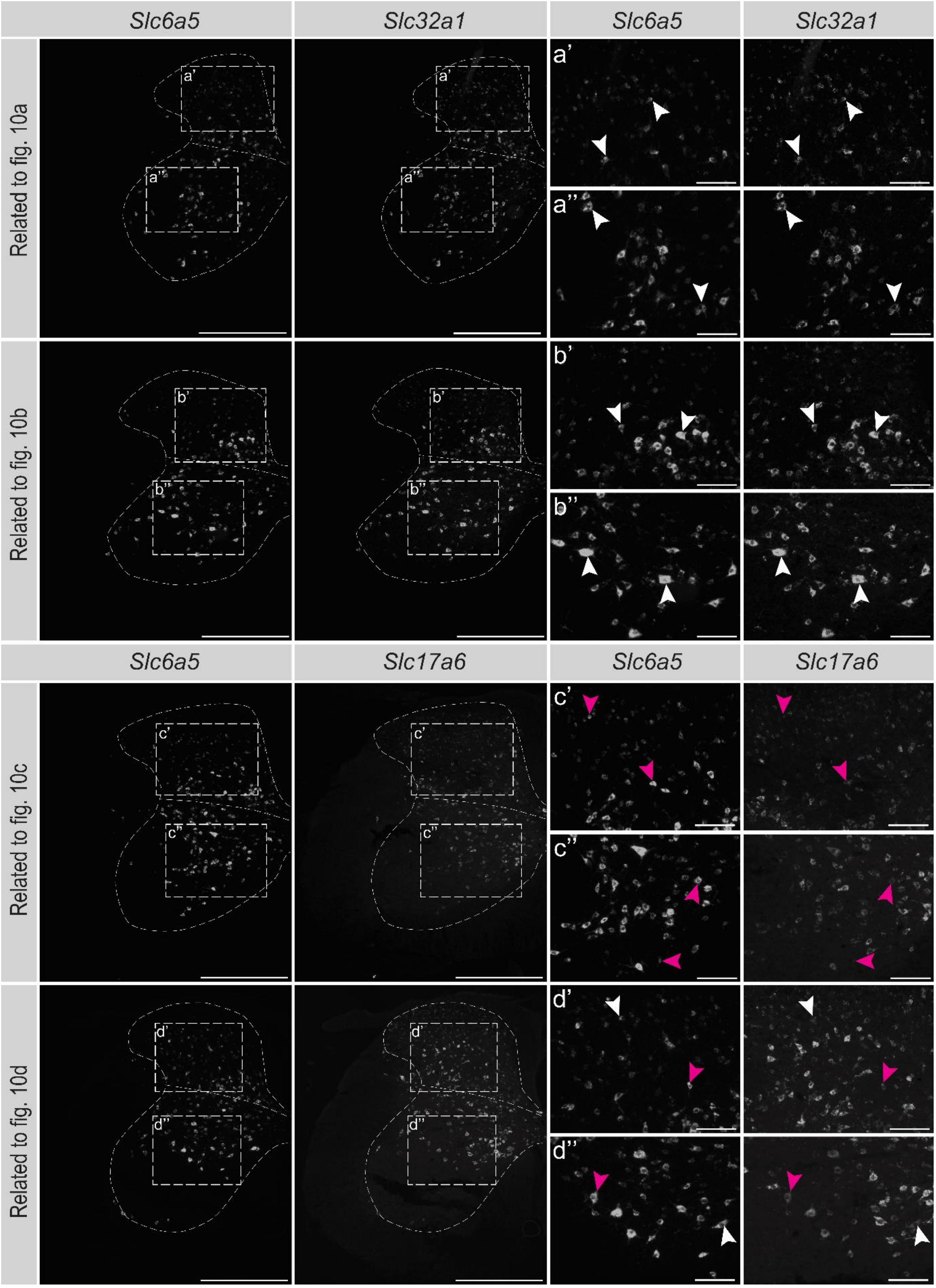


**Fig S10, relates to fig 10.** *Histological analysis of the expression of Slc6a5 in the lumbar spinal cord.* Fluorescent RNAscope for *Slc6a5* was combined with *Slc32a1* (inhibitory marker)

or *Slc17a6* (excitatory marker). The figure displays the separate channels for *Slc6a9, Slc32a1 and Slc17a6* in (grey) in the lumbar section of the spinal cord. Dashed boxes show the position of the enlargement. White arrows indicate examples of double-positive cells, while magenta arrows highlight examples of cells expressing *Slc6a9* only (≥ 5 dots within the same cell), the dashed boxes and arrows display the same areas and cells indicated in Fig. 10a'–d''). Faint background staining can be seen in the *Slc17a6* channel. However, as no RNAscope dots can be observed, the signal stems from bleed-through from the *Slc6a5* channel.

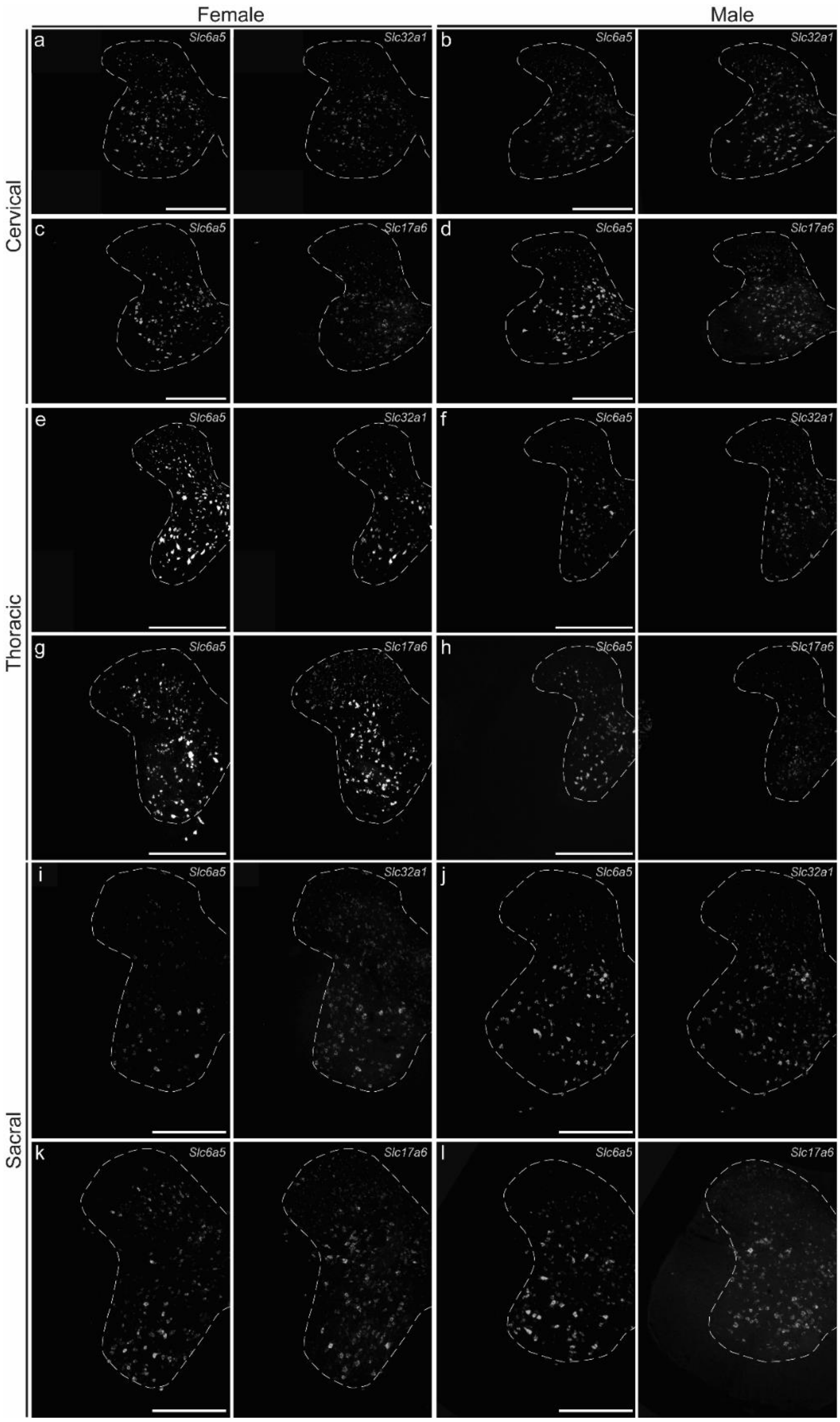


**Fig S11, relates to fig 10 and Additional file 1: Fig. S7–9.** *Histological analysis of the expression of Slc6a5 in the cervical, thoracic and sacral spinal cord.* Fluorescent RNAscope for *Slc6a5* was combined with *Slc32a1* (inhibitory marker) or *Slc17a6* (excitatory marker). The figure displays the separate channels for *Slc6a9, Slc32a1 and Slc17a6* in (grey).

Table S1, relates to figure 3: *$Log_2$ fold differences in Slc6a9 and Slc6a5 expression across nervous system regions and peripheral organs relative to background controls.* Statistical analysis was performed using the Kruskal–Wallis (KW) test followed by Dunn’s multiple comparisons test to evaluate expression relative to background levels in the qRT-PCR runs. Comparisons were conducted separately for females (n = 5) and males (n = 5). Calculations were performed both with and without outliers; values in parentheses indicate the calculated p-value with outliers included.

| Tissue | *Slc6a9* | | | | *Slc6a5* | | | |
|---|---|---|---|---|---|---|---|---|
| | Females | | Males | | Females | | Males | |
| | KW | Dunn’s | KW | Dunn’s | KW | Dunn’s | KW | Dunn’s |
| Cortex | <0.0001 | 0.0856 | <0.0001 | >0.9999 | <0.0001 | >0.9999 | <0.0001 | 0.0727 |
| Amygdala | | >0.9999 | | 0.3814 | | 0.4659 | | >0.9999 |
| Striatum | | 0.0002 | | 0.4139 | | >0.9999 | | 0.0232 |
| Hypothalamus | | 0.0259 | | 0.1694 | | >0.9999 | | >0.9999 |
| Thalamus | | 0.0029 | | <0.0001 | | >0.9999 | | 0.2858 |
| Hippocampus | | >0.9999 | | 0.4607 | | >0.9999 | | >0.9999 |
| Cerebellum | | 0.0003 | | 0.0064 | | 0.1167 | | 0.0012 |

| | | | | | | | | |
|---|---|---|---|---|---|---|---|---|
| Brainstem | | 0.0038 | | 0.0031 | | 0.0150 | | 0.0002 |
| Spinal cord | | 0.0014 | | <0.0001 | | 0.0425 | | <0.0001 |
| | | | | | | | | |
| Thymus | 0.0016 (0.0284) | 0.0419 | 0.0002 | >0.9999 | 0.1542 (0.1843) | 0.0890 | 0.0754 (0.1280) | 0.7156 (0.0874) |
| Lung | | 0.0128 | | >0.9999 | | >0.9999 | | 0.1404 (0.0689) |
| Liver | | 0.6604 | | 0.0026 | | >0.9999 | | 0.4991 (0.2537) |
| Intestine | | >0.9999 (>0.9999) | | 0.2682 | | >0.9999 | | 0.1100 (0.1603) |
| Spleen | | 0.0145 (>0.9999) | | 0.1359 | | >0.9999 (0.2525) | | 0.4991 (0.0746) |
| Kidney | | >0.9999 | | 0.3391 | | 0.1229 | | >0.9999 (0.2133) |

| | | | | | | | | |
|---|---|---|---|---|---|---|---|---|
| Genitalia | | 0.0747 (0.6278) | | >0.9999 | | 0.1808 | | >0.9999 (0.5165) |

## References


1. Zeisel A, Hochgerner H, Lonnerberg P, Johnsson A, Memic F, van der Zwan J, et al. Molecular Architecture of the Mouse Nervous System. Cell. 2018;174(4):999-1014 e22.
2. Vandesompele J, De Preter K, Pattyn F, Poppe B, Van Roy N, De Paepe A, et al. Accurate normalization of real-time quantitative RT-PCR data by geometric averaging of multiple internal control genes. Genome Biol. 2002;3(7):RESEARCH0034.
